# Antagonism Between DUX4 and DUX4c Highlights a Pathomechanism Operating Through β-Catenin in Facioscapulohumeral Muscular Dystrophy

**DOI:** 10.3389/fcell.2022.802573

**Published:** 2022-09-07

**Authors:** Massimo Ganassi, Nicolas Figeac, Magalie Reynaud, Huascar Pedro Ortuste Quiroga, Peter S. Zammit

**Affiliations:** Randall Centre for Cell and Molecular Biophysics, King’s College London, London, United Kingdom

**Keywords:** DUX4, DUX4c, proliferation, FSHD, facioscapulohumeral muscular dystrophy, β-CATENIN, DUX4L9, WNT signalling

## Abstract

Aberrant expression of the transcription factor DUX4 from D4Z4 macrosatellite repeats on chromosome 4q35, and its transcriptome, associate with pathogenesis in facioscapulohumeral muscular dystrophy (FSHD). Forced DUX4 expression halts skeletal muscle cell proliferation and induces cell death. DUX4 binds DNA via two homeodomains that are identical in sequence to those of DUX4c (DUX4L9): a closely related transcriptional regulator encoded by a single, inverted, mutated D4Z4 unit located centromeric to the D4Z4 macrosatellite array on chromosome 4. However, the function and contribution of DUX4c to FSHD pathogenesis are unclear. To explore interplay between DUX4, DUX4c, and the DUX4-induced phenotype, we investigated whether DUX4c interferes with DUX4 function in human myogenesis. Constitutive expression of DUX4c rescued the DUX4-induced inhibition of proliferation and reduced cell death in human myoblasts. Functionally, DUX4 promotes nuclear translocation of β-CATENIN and increases canonical WNT signalling. Concomitant constitutive expression of DUX4c prevents β-CATENIN nuclear accumulation and the downstream transcriptional program. DUX4 reduces endogenous DUX4c levels, whereas constitutive expression of DUX4c robustly suppresses expression of DUX4 target genes, suggesting molecular antagonism. In line, DUX4 expression in FSHD myoblasts correlates with reduced DUX4c levels. Addressing the mechanism, we identified a subset of genes involved in the WNT/β-CATENIN pathway that are differentially regulated between DUX4 and DUX4c, whose expression pattern can separate muscle biopsies from severely affected FSHD patients from healthy. Finally, blockade of WNT/β-CATENIN signalling rescues viability of FSHD myoblasts. Together, our study highlights an antagonistic interplay whereby DUX4 alters cell viability via β-CATENIN signalling and DUX4c counteracts aspects of DUX4-mediated toxicity in human muscle cells, potentially acting as a gene modifier for FSHD severity. Importantly, direct DUX4 regulation of the WNT/β-CATENIN pathway informs future therapeutic interventions to ameliorate FSHD pathology.

## Introduction

Facioscapulohumeral muscular dystrophy (FSHD) is the third most common muscular dystrophy, manifesting as a descending left/right asymmetric muscular weakness and wasting. FSHD is slowly progressive, initially generally affecting facial, shoulder, and proximal upper limb muscles and continuing to lower limb muscles (Greco et al., 2020). In addition to dystrophic musculature, FSHD symptoms can also include extra-muscular manifestations such as sensorineural hearing loss and retinal vasculopathy, indicating a complex underlying molecular pathogenesis (Banerji and Zammit, 2021).

FSHD is an autosomal-dominant condition, with *de novo* cases less frequent. FSHD is associated with epigenetic derepression occurring at the subtelomeric region of chromosome 4 (4q35), covering a large macrosatellite array of D4Z4 repeats that are normally transcriptionally silenced through epigenetic mechanisms (Wijmenga et al., 1992; van Deutekom et al., 1993; van Overveld et al., 2003). FSHD aetiology is classified into two groups. FSHD1 (OMIM: 158900) is the more prominent (95% of cases) where the 4q35 locus has a reduction of D4Z4 units from the usual ≥11–100+ copies found in non-affected individuals, to only 1–10 repeats on at least one allele in FSHD1 patients (Wijmenga et al., 1992; Hewitt et al., 1994). In contrast, the remaining 5% of cases are classified as FSHD2 (OMIM: 158901), where the residual number of D4Z4 units is usually within the lower end of the ‘normal’ range, and epigenetic derepression is primarily caused by mutations in the chromatin remodelling protein SMCHD1 (Lemmers et al., 2012; van den Boogaard et al., 2016). Each 3.3 kb D4Z4 unit contains an open reading frame encoding a transcription factor called Double Homeobox 4 (DUX4). Epigenetic derepression in FSHD allows transcription of the DUX4 retrogene from the distal-most D4Z4 unit, with the mRNA stabilised for translation by a polyadenylation signal located in the flanking DNA of permissive 4qA haplotypes (Dixit et al., 2007; Lemmers et al., 2010). Thus, mis-expression of DUX4 is strongly associated with FSHD pathogenesis (Lim et al., 2020; Banerji and Zammit, 2021).

In muscle cells, ectopic DUX4 expression rapidly cascades into alterations in many cellular processes, including inhibition of cell-cycle progression and myogenic differentiation, to promotion of cell death through CASPASE-mediated apoptosis (Himeda and Jones, 2019). DUX4 regulates many target genes and amongst the myriad of signalling pathways affected by DUX4 and dysregulated in FSHD, is the WNT/β-CATENIN interactome, nodal to vertebrate myoblast proliferation and differentiation (Schmidt et al., 2000; Fitzsimons, 2011; Banerji et al., 2015; Rudnicki and Williams, 2015; Suzuki et al., 2015). CASPASE3 and β-CATENIN signalling strongly correlate in FSHD muscle, suggesting that such alterations contribute to loss of cell homeostasis (Banerji et al., 2015). Importantly, blocking β-CATENIN degradation suppresses DUX4 expression and prevents apoptosis in differentiated muscle cells (Block et al., 2013), highlighting a role for β-CATENIN in DUX4-mediated toxicity, and suggesting modulation via negative feedback. However, how DUX4 affects β-CATENIN signalling is unresolved.

Besides permitting DUX4 expression, epigenetic derepression at D4Z4 could also alter/activate expression of nearby genes located centromeric to 4q35. The so-called 4qter genes include *DUX4c* (*DUX4L9*) (Ansseau et al., 2009), *FRG1* (Gabellini et al., 2002), *FRG2* (Rijkers et al., 2004), *TUBB4Q* (Van Geel et al., 2000), and *ANT1* (*SLC25A4*) (Doerner et al., 1997).

*DUX4c* is encoded by a single truncated and inverted D4Z4 unit located 42 kb centromeric to the D4Z4 array on chromosome 4 (Ansseau et al., 2009). There is high sequence similarity between DUX4 and DUX4c over most of the encoded proteins, including the two DNA-binding homeodomains which are identical, although there is divergence in the C-terminal region due to a nonsense mutation causing a truncation in DUX4c (Bosnakovski et al., 2008a; Ansseau et al., 2009). DUX4c is detectable in FSHD muscle biopsies and proliferating FSHD myoblasts, and increases upon myogenic differentiation, in line with global epigenetic activation of 4qter genes (Ansseau et al., 2009). DUX4c has mixed effects on proliferation depending on the study either not affecting viability of human myoblasts, or increasing the proliferative capacity of human rhabdomyosarcoma cells, but reducing that of murine myoblasts (Bosnakovski et al., 2008a; Ansseau et al., 2009; Knopp et al., 2016; Bosnakovski et al., 2018). Moreover, DUX4c alters expression of myogenic genes and inhibits progression of myogenic differentiation (Bosnakovski et al., 2008a; Ansseau et al., 2009; Knopp et al., 2016; Vanderplanck et al., 2018), highlighting functional overlap with DUX4 but also suggesting differences in temporal activation during myogenesis. Previous transcriptomic analysis highlighted that DUX4 and DUX4c not only induce both unique and overlapping transcriptional changes but might also exert transcriptional inhibition on a proportion of genes (Banerji et al., 2015; Dmitriev et al., 2016; Knopp et al., 2016). Intriguingly, a very small fraction (0.6%) of FSHD patients bear a genomic deletion encompassing the *DUX4c* locus, indicating that DUX4c does not cause FSHD (Lemmers et al., 2003; Deak et al., 2007), but DUX4c could instead modulate DUX4 function in FSHD. However, molecular interplay between DUX4 and DUX4c in FSHD pathogenesis is under studied.

The prevailing model of FSHD is that aberrant expression of DUX4 is the root cause of FSHD. In fact, much preclinical and clinical endeavor is directed at suppressing ongoing DUX4 expression as a therapy, although DUX4 mRNA/protein are notoriously difficult to detect in both FSHD patient-derived muscle cells and post-natal muscle biopsies. Thus, identification of alternative approaches to tackle effects of DUX4-mediated toxicity remains an urgent priority, such as addressing the suppression of PAX7 target genes that characterises FSHD (Banerji et al., 2017; Banerji and Zammit, 2021).

Here, we explore aspects of the DUX4-induced phenotype and assess the ability of DUX4c to modulate them. Constitutive expression of DUX4c in a DUX4-inducible human myoblast model inhibits DUX4-mediated reduction in cell proliferation, and efficiently decreases cell death. Mechanistically, DUX4 promotes β-CATENIN nuclear translocation and its subsequent transactivation of target genes. However, inhibition of β-CATENIN activity significantly blunts DUX4-induced apoptosis, indicating that DUX4 toxicity involves the canonical WNT/β-CATENIN pathway. Likewise, expression of DUX4c in DUX4-expressing myoblasts reduces nuclear β-CATENIN and activation of target genes, unraveling a DUX4/DUX4c molecular antagonism converging on cell viability. DUX4 accumulation reduces endogenous *DUX4c* expression whereas constitutive expression of DUX4c robustly decreases activation of DUX4 target genes, confirming DUX4/DUX4c interplay. Transcriptomic analysis reveals a subset of WNT/β-CATENIN genes that are differentially regulated by DUX4 and DUX4c that discriminate between severely affected FSHD and healthy muscle biopsies, suggesting a minimal biomarker signature conserved in FSHD muscle. In support, patient-derived FSHD myoblasts display significantly reduced DUX4c levels in parallel to higher DUX4 expression, compared to matched controls. FSHD myoblasts have a reduced proliferation rate, which can be rescued by the inhibition of β-CATENIN. Together, our study demonstrates that DUX4-mediated toxicity in human myoblasts involves canonical WNT/β-CATENIN signalling, which can be counteracted by DUX4c. This implies an underlying DUX4/DUX4c molecular antagonism in FSHD, with DUX4c acting as a gene modifier for pathogenesis.

## Results

### DUX4 Inhibits Proliferation and Induces Cell Death in Human Myoblasts

**FIGURE 1:**
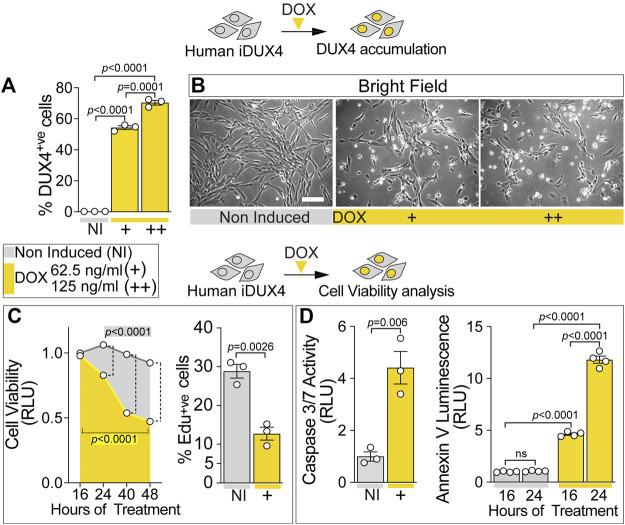
DUX4 blocks proliferation and triggers cell death in human myoblasts. **(A)** Percentage of iDUX4 myoblasts containing DUX4 (DUX4^+ve^) showing dose-dependent effect of Doxycycline (DOX) (+: 62.5 ng/ml; ++: 125 ng/ml) for 24 h compared to non-induced control (NI), N = 3 biological replicates, unpaired t-test. **(B)** Representative bright field images of proliferating human iDUX4 myoblasts induced with 62.5 ng/ml (+) or 125 ng/ml (++) DOX for 24 h, showing reduction in cell viability upon DUX4 accumulation. Scale bar equals 100 µm. **(C)** Quantification of cellular viability following DUX4 induction (+: 62.5 ng/ml DOX) shows persistent decline over time compared to non-induced iDUX4 cells (left). N = 4 biological replicates, ANOVA, Tukey’s posthoc test for viability. Highlighted *p* values indicate significance between 16 and 48 h of DOX treatment (yellow) or in overall viability trend between non-induced and induced cells. Vertical dashed lines indicate *p* values < 0.0001 between non-induced and induced samples at given time points. RLU; Relative Luciferase Units reported as fold change to non-induced (NI) 16 h sample. Percentage of iDUX4 myoblasts that had incorporated EdU (EdU^+ve^) was significantly reduced upon 24 h of DUX4 induction (right). N = 3 biological replicates for EdU, unpaired t-test. +: 62.5 ng/ml DOX. **(D)** Quantification of Caspase3/7 activity upon DOX treatment shows significant increase in DUX4-induced cells (+: 62.5 ng/ml) compared to non-induced control (left). N = 3 biological replicates, unpaired t-test for Caspase 3/7. DUX4 accumulation leads to increased apoptosis over time, as shown by increased Annexin V (right). N = 4 biological replicates, ANOVA, Tukey’s posthoc test. RLU; Relative Luciferase Units reported as fold change to non-induced (NI) 16 h sample. Graphs report mean ± SEM from representative experiments. Statistical significance between specific samples indicated by a bar.

To standardise DUX4 induction in human LHCN-M2-iDUX (iDUX4) myoblasts (Choi et al., 2016), we administered increasing concentrations of doxycycline (DOX) for 24 hours (h). This led to a dose-dependent increase in the proportion of myoblasts containing DUX4 (Figure 1A; Supplementary Figures S1A–D). Notably, the level of *DUX4* mRNA was significantly increased after 7 h of high-dose DOX treatment (250 ng/ml), whereas expression of DUX4 target genes *TRIM43*, *PRAMEF1*, *ZSCAN4*, and *KHDC1L* peaked between 16–24 h of DOX treatment, when *DUX4* mRNA levels were reduced compared with the 7 h time point. Thus, robust activation of DUX4-target genes starts after approximately 9–10 h of DOX induction (Supplementary Figure S1E). We selected 24 h of DUX4 induction for analysis.

iDUX4 cell number was significantly decreased upon DUX4 induction using 62.5 ng/ml DOX or higher (Figure 1B; Supplementary Figure S1D), suggesting that DUX4 accumulation reduces cell viability. Indeed, treatment of iDUX4 with either 62.5 (low dose) or 250 ng/ml (high dose) DOX significantly reduced cell viability compared with untreated iDUX4 control cells (Figure 1C; Supplementary Figure S1F). Since high-dose DOX treatment did not have major additional effects, 62.5 ng/ml was selected for further analysis (Supplementary Figure S1F).

To explore proliferation dynamics upon DUX4 expression, iDUX4 myoblasts were pulsed with EdU for 2 h after 24 h in growth medium supplemented with DOX, and EdU incorporation compared with non-induced iDUX4 (Figure 1C; Supplementary Figure S1G). The proportion of cells in S-phase decreased significantly upon DUX4 accumulation. Moreover, EdU incorporation was inversely correlated with increasing concentrations of DOX (Supplementary Figure S1G), mirroring the increment in DUX4-positive cells (Figure 1A; Supplementary Figure S1C). Thus, iDUX4 myoblast proliferation rate was significantly reduced by DUX4 up-regulation induced by ≥62.5 ng/ml of DOX (Supplementary Figure S1G).

DUX4 accumulation caused altered cell morphology, inducing a smaller and irregular cell shape phenotype, likely indicating the onset of programmed cell death (Figure 1B; Supplementary Figure S1H) in line with previous reports (Kowaljow et al., 2007; Choi et al., 2016). Congruent with the initiation of apoptosis, 16 h of induced DUX4 expression was sufficient to significantly augment Caspase3/7 activity, confirming the onset of the apoptotic program (Figure 1D). Analysis of apoptosis using membrane-exposed phosphatidylserine (Martin et al., 1995) revealed a significant increase already after 16 h from the DUX4 induction, further increasing at 24 h (Figure 1D). As seen for proliferation, treatment with higher dose of DOX did not enhance cell death (Supplementary Figure S1H). Notably, the apoptotic process was accompanied by detectable necrosis, as determined by loss of cell membrane integrity (Supplementary Figure S1H). Thus, DUX4 accumulation reduces human myoblast viability in a dose-dependent manner.

### DUX4c Rescues DUX4-Mediated Proliferation Defect and Reduces Cell Death

Several lines of evidence indicate common mechanistic features between DUX4 and DUX4c due to the high similarity over most of the protein sequence (Bosnakovski et al., 2008a; Ansseau et al., 2009; Bosnakovski et al., 2009; Dmitriev et al., 2016; Knopp et al., 2016), suggesting that DUX4-DUX4c interplay may contribute to FSHD pathogenesis. To assess the effect of DUX4c on the DUX4-induced phenotype, human iDUX4 myoblasts were transduced with retrovirus encoding either DUX4c and IRES-eGFP (RV_DUX4c-IRES-eGFP) or the control retroviral backbone with just IRES-eGFP (RV_-IRES-eGFP) (Knopp et al., 2016) and stable iDUX4 lines made (schematic in Supplementary Figure S2) constitutively expressing either DUX4c-IRES-eGFP (iDUX4/DUX4c) or just IRES-eGFP (iDUX4/Ctrl). Immunolabelling of transduced but non-induced iDUX4/DUX4c myoblasts confirmed accumulation of DUX4c protein (Supplementary Figure S2A). RT-qPCR analysis confirmed significant up-regulation of *DUX4c* mRNA in iDUX4/DUX4c non-induced myoblasts, compared with iDUX4/Ctrl (Supplementary Figure S2B).

**FIGURE 2:**
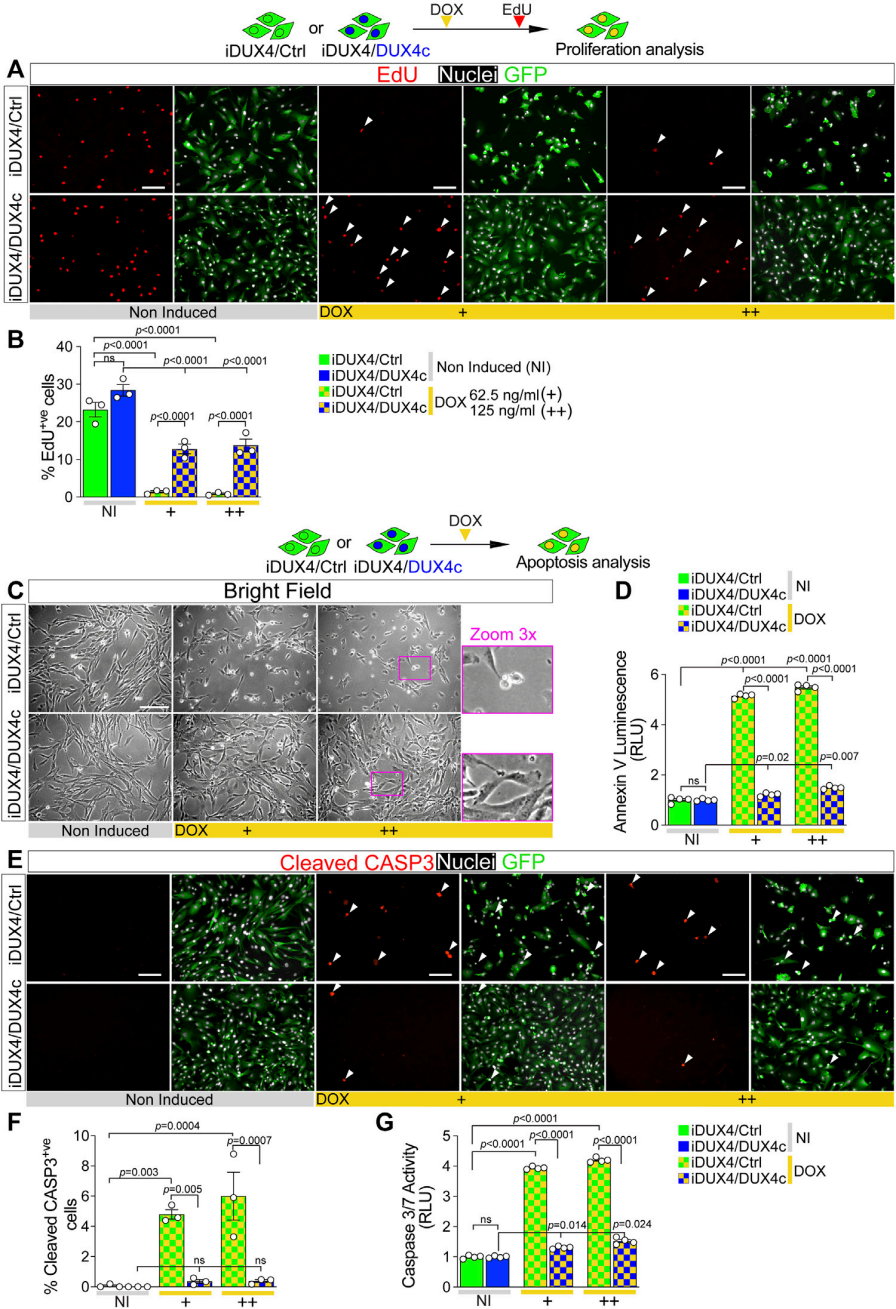
DUX4c prevents DUX4 toxicity in proliferating myoblasts. **(A)** Representative images of proliferating iDUX4/Ctrl or iDUX4/DUX4c myoblasts when non-induced or induced with either 62.5 ng/ml (+) or 125 ng/ml (++) DOX for 24 h and immunolabelled for GFP (green), with EdU incorporation visualised (red-arrowheads) and nuclei counterstained with DAPI (white). Scale bar equals 100 µm. **(B)** The percentage of iDUX4/Ctrl or iDUX4/DUX4c myoblasts that incorporated EdU (EdU^+ve^) shows that constitutive expression of DUX4c significantly reduces the anti-proliferative effect of DUX4 compared to RV-ctrl. N = 3 biological replicates, ANOVA, Tukey’s posthoc test. **(C)** Representative images showing that DUX4 up-regulation (24 h of 62.5 ng/ml DOX (+) or 125 ng/ml (++)) results in accumulation of more apoptotic cells in DOX-induced iDUX4/Ctrl than in iDUX4/DUX4c myoblasts, with non-induced (NI) iDUX4 shown for comparison. Scale bar represents 100 µm. **(D)** Quantification of apoptosis (Annexin V Luminescence) on iDUX4 myoblasts when non-induced (NI) or after 62.5 ng/ml DOX (+) or 125 ng/ml (++) for 24 h shows significant induction of apoptosis in iDUX4/Ctrl, but not in iDUX4/DUX4c myoblasts, compared to non-induced (NI) controls. RLU; Relative Luciferase Units reported as fold change to non-induced (NI) iDUX4/Ctrl. N = 4 biological replicates, ANOVA, Tukey’s posthoc test. **(E)** Representative images of proliferating iDUX4/Ctrl and iDUX4/DUX4c myoblasts induced with either 62.5 (+) or 125 ng/ml (++) DOX for 24 h and co-immunolabelled for Cleaved-CASPASE3 (CASP3) (red) and GFP (green), with DAPI counterstained nuclei (white). Arrowheads indicate Cleaved-CASP3^+ve^ nuclei in DUX4-induced samples. Scale bar equals 100 µm. **(F)** DUX4-induction increased the percentage of cleaved-CASP3-positive (CASP3^+ve^) iDUX4/Ctrl myoblasts, but not in iDUX4/DUX4c cells. N = 3 biological replicates, ANOVA, Tukey’s posthoc test. **(G)** Quantification of Caspase3/7 activity in iDUX4 myoblasts treated as in F shows significant induction of Caspase3/7 activity in DUX4-induced iDUX4/Ctrl, but not in DUX4-induced iDUX4/DUX4c myoblasts, compared to non-induced (NI) controls. RLU; Relative Luciferase Units reported as fold change to non-induced (NI) iDUX4/Ctrl. N = 4 biological replicates, ANOVA, Tukey’s posthoc test. Graphs report mean ± SEM from representative experiments. Statistical significance between specific samples indicated with bars.

To test effects of concomitant expression of DUX4c with DUX4, iDUX4/Ctrl and iDUX4/DUX4c myoblasts were cultured in growth medium and DUX4 expression was DOX-induced for 24 h prior to a 2 h EdU pulse. DUX4c expression did not affect the proliferation rate in non-induced iDUX4/DUX4c myoblasts (Figures 2A,B). As expected, up-regulation of DUX4 led to significant reduction in the proliferation rate in control iDUX4/Ctrl myoblasts at both 62.5 and 125 ng/ml of DOX (Figures 2A,B), consistent with effects in untransduced iDUX4 cells (Figure 1). Strikingly, constitutive expression of DUX4c significantly reduced the effect of DUX4 on proliferation in iDUX4/DUX4c myoblasts, irrespective of DOX dose (Figures 2A,B). Therefore, DUX4c efficiently attenuates the DUX4-mediated reduction in the proliferation rate.

The anti-proliferative effect of DUX4 is accompanied by the onset of apoptotic events (Kowaljow et al., 2007; Block et al., 2013; Rickard et al., 2015) (Figure 1D), so we examined whether DUX4c could also mitigate DUX4-driven cytotoxicity. DUX4c expression did not cause apoptosis in non-induced iDUX4/DUX4c myoblasts, as shown by cell morphology and an assay for Annexin V (Figures 2C,D). 24 h DUX4 induction led to appearance of rounded iDUX4/Ctrl myoblasts (Figure 2C), confirming previous observations (Supplementary Figure S1H). In contrast, iDUX4/DUX4c myoblast morphology was mostly unaffected by DUX4 up-regulation (Figure 2C), suggesting a protective effect of DUX4c. Indeed, the Annexin V assay revealed a significant increase in apoptosis after 24 h from the DUX4 induction in iDUX4/Ctrl (Figure 2D) but not in iDUX4/DUX4c myoblasts, which instead displayed resistance to DUX4-induced apoptosis (Figure 2D).

Activation of CASPASE3 contributes to DUX4 toxicity (Wallace et al., 2011; Knopp et al., 2016; DeSimone et al., 2019). To further assess the ability of DUX4c to suppress DUX4-induced initiation of programmed cell death, we analysed accumulation of cleaved CASPASE3 (cCASP3) using immunolabelling and Caspase activity upon DUX4 up-regulation (Figures 2E–G). DUX4c did not affect either accumulation of cCASP3 or Caspase3/7 activity in non-induced iDUX4/DUX4c myoblasts (Figures 2E–G). DUX4 induction led to a significant increase in the proportion of DOX-induced iDUX4/Ctrl myoblasts with cCASP3 and Caspase activity (Figures 2E,F), confirming induction of apoptosis. However, concomitant expression of DUX4c prevented cCASP3 accumulation in DOX-induced iDUX4/DUX4c myoblasts (Figures 2E,F). DUX4c also drastically attenuated the increase in Caspase activity in DOX-induced iDUX4/DUX4c, compared with iDUX4/Ctrl, myoblasts (Figure 2G). Therefore, DUX4c dramatically diminishes DUX4-induced CASP3-mediated apoptosis and reduction of cell viability.

### DUX4/DUX4c Transcriptional Signatures Converge on WNT/β-CATENIN Signalling

**FIGURE 3:**
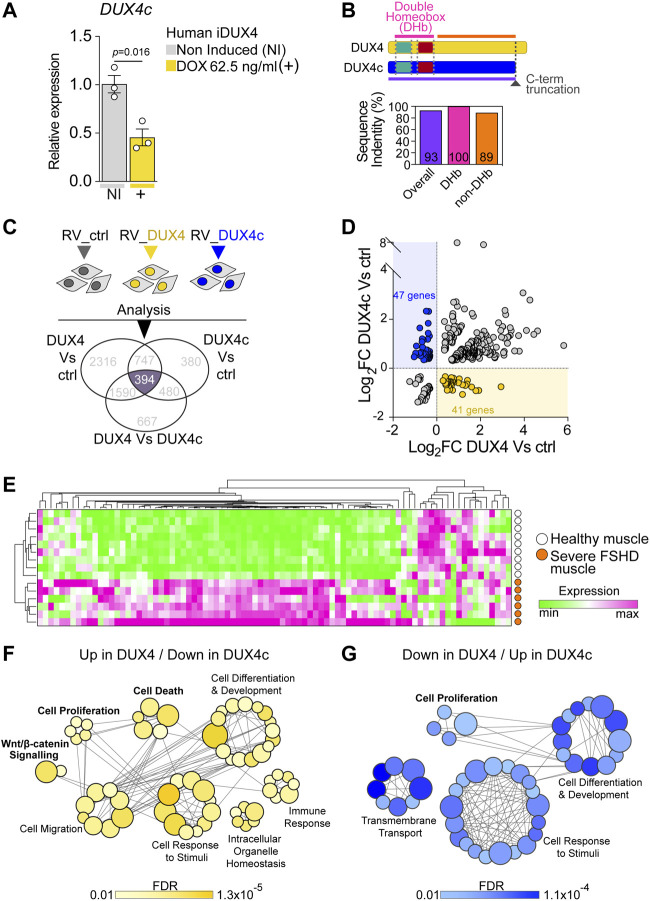
DUX4 and DUX4c transcriptomes converge on cell homeostasis in FSHD. **(A)** RT-qPCR analysis on iDUX4 myoblasts when non-induced or after treatment with 62.5 ng/ml DOX (+) for 24 h showing down-regulation of *DUX4c* transcripts. Graph reports mean ± SEM, N = 3 biological replicates, unpaired two-tailed t-test. **(B)** Schematic of DUX4 and DUX4c proteins depicting the two identical homeobox domains and the C-terminal truncation (dashed dark grey line) in DUX4c compared to DUX4. Graph reports the amino acid sequence identity of DUX4 and DUX4c: either overall, just over the two homeobox domains (DHb), or the more C-term sequence (non-DHb). Percentage identity is reported within columns which are colour-coded with lines in the protein schematic above. **(C)** Venn diagram illustrating transcriptomic analysis of murine primary satellite cell-derived myoblasts transduced with human DUX4 (RV_DUX4), DUX4c (RV_DUX4c), or control retroviral vector (RV_ctrl) reveal a common set of 394 differentially expressed mouse probes (referring to 356 human ortholog genes). Expression values retrieved from GSE77100 Banerji et al. (2015), Knopp et al. (2016). **(D)** Volcano plot depicting that 88 human ortholog genes display opposite regulation by DUX4 and DUX4c in murine myoblasts. 41/356 are up-regulated by DUX4/down-regulated by DUX4c (yellow dots) while 47/356 are down-regulated by DUX4/up-regulated by DUX4c (blue dots). **(E)**. Expression of these 88 genes is sufficient to separate severe (group 4) human FSHD (*n* = 6, orange) and healthy muscle (*n* = 9, white) biopsies upon hierarchical clustering analysis. Expression values retrieved from GSE115650 (Wang et al., 2019). **(F)** Gene Ontology (GO) analysis retrieved by the subset of 41 target genes up-regulated by DUX4/down-regulated by DUX4c. **(G)** Gene Ontology (GO) analysis retrieved by the subset of 47 target genes down-regulated by DUX4/up-regulated by DUX4c. Main biological processes enriched by either subset are indicated with bubbles representing specific GOs, coloured based on False Discovery Rate (FDR) values, and size proportional to number of genes within specific GO terms, grey lines represent genes shared across different GOs.

The DUX4c effect on the DUX4-induced phenotype is in line with previous transcriptomic profiling on murine satellite cell-derived myoblasts. Transduction with either human DUX4 or DUX4c indicates that DUX4c might repress a considerable fraction of DUX4 target genes, suggesting that DUX4c could act in an antagonistic manner to DUX4 (Banerji et al., 2015; Knopp et al., 2016). To gain a mechanistic insight into the interplay between DUX4 and DUX4c, we first examined whether induction of DUX4 alters expression of endogenous DUX4c. RT-qPCR analysis showed that DUX4 up-regulation following 24 h with 62.5 ng/ml DOX in iDUX4 myoblasts led to a significant decrease in *DUX4c* transcripts (Figure 3A), suggesting that DUX4 negatively regulates *DUX4c* accumulation.

Since the antagonistic transcriptomic intersection likely arises from the 100% identity between the amino acid sequence of the DUX4 and DUX4c homeodomains (Figure 3B; Supplementary Figures S3A,B), we evaluated the extent of transcriptional overlap by considering all genes differentially expressed among murine satellite cell-derived myoblasts, transduced with either DUX4, DUX4c or the control retroviral backbone using a publicly available dataset (GSE77100) (Banerji et al., 2015; Knopp et al., 2016). Transcriptomic analysis retrieved 394 differentially expressed murine probes (Figure 3C), which referred to 356 human ortholog genes.

To explore antagonism between DUX4 and DUX4c, we focussed on the 356 human ortholog genes that were differentially expressed in murine myoblasts that were also regulated in opposite directions by DUX4 and DUX4c. We found that 41 genes were up-regulated by DUX4 and down-regulated by DUX4c, whereas 47 genes were down-regulated by DUX4 and up-regulated by DUX4c (Figure 3D), totalling 88/356 (25%) genes (Supplementary Table S2). Thus, DUX4 and DUX4c exert opposite transcriptional regulation on a subset of genes in murine myoblasts, underlying an antagonistic interplay between the two transcription factors.

Next, we tested the ability of this 88-gene transcriptional signature to discriminate between muscle biopsies from patients with severe FSHD or healthy subjects using a publicly available dataset (GSE115650) (Wang et al., 2019). Intriguingly, the expression profile of the 88 genes efficiently clustered FSHD samples separately from healthy controls upon hierarchical clustering analysis (Figure 3E; Supplementary Table S3), strengthening the hypothesis that DUX4/DUX4c antagonism may contribute to FSHD pathomechanisms. Interestingly, the majority of the genes in the signature displayed a trend toward up-regulation (Figure 3E) in severe FSHD muscle, possibly arising from the heterogeneous cell populations in muscle biopsies (e.g., myogenic, immune, vascular, etc.).

To better investigate the candidate biomarker signature, we then interrogated the 41 Up in DUX4/Down in DUX4c and 47 Down in DUX4/Up in DUX4c target gene subsets using Gene Ontology (GO) analysis to evaluate their contribution to specific biological processes. The two gene subsets retrieved the common processes ‘Cell Differentiation and Development’ and ‘Cell Response to Stimuli’. However, the Up in DUX4/Down in DUX4c geneset-specific biological processes included ‘WNT/β-CATENIN’, ‘Immune Response’, and ‘Cell Death’ (Figure 3F), while the Down in DUX4/Up in DUX4c returned ‘Cell Proliferation’ and ‘Transmembrane Transport’ (Figure 3G). Given the observed rescue at the cellular level of proliferation and cell death upon DUX4c constitutive expression in DUX4-induced iDUX4/DUX4c myoblasts (Figure 2), we conclude that the ability of DUX4c to counteract DUX4-toxicity is likely to arise from DUX4/DUX4c transcriptional antagonism regulating myoblast homeostasis, in parallel to DUX4 suppressing DUX4c expression.

### DUX4 Promotes Nuclear Location of β-CATENIN and Activation of WNT/ β-CATENIN Target Genes

**FIGURE 4:**
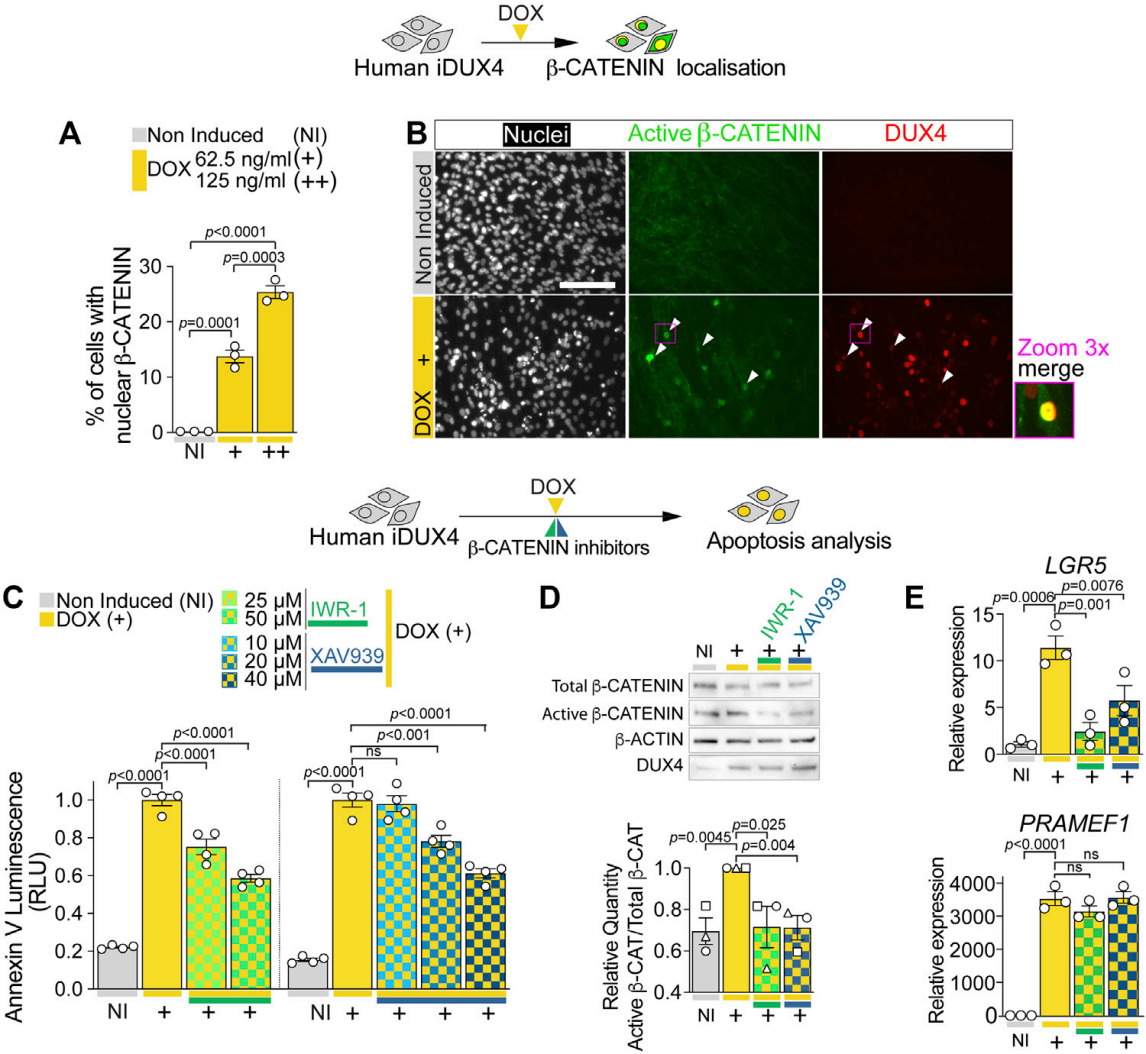
DUX4 increases β-CATENIN nuclear shuttling and activity. **(A)** Quantification of the percentage of iDUX4 myoblasts with nuclear β-CATENIN when non-induced (NI) or after treatment with either 62.5 (+) or 125 ng/ml (++) DOX for 24 h showing dose-dependent effect of DOX compared to non-induced control. N = 3 biological replicates, ANOVA, Tukey’s posthoc test. **(B)** Representative images of proliferating non-induced iDUX4 myoblasts and those induced with 62.5 ng/ml DOX (+) for 24 h and immunolabelled for Active β-CATENIN (green) and DUX4 (red) with nuclei counterstained with DAPI (white) showing nuclear accumulation upon DUX4 induction. Arrowheads indicate Active β-CATENIN localisation in induced cells. Scale bar represents 100 µm. **(C)** Quantification of apoptosis in iDUX4 myoblasts when non-induced (NI) or after 62.5 ng/ml DOX (+) for 24 h shows robust apoptosis upon DOX treatment, that is significantly reduced when co-treated with indicated β-CATENIN inhibitors at shown concentration, compared to non-induced (NI) controls. Coloured lines beneath graphs indicate specific inhibitors. N = 4 biological replicates, ANOVA, Tukey’s posthoc test. **(D)** Representative Western blot and quantification in proliferating non-induced iDUX4 myoblasts (NI) and those induced with either 62.5 ng/ml DOX (+) or 62.5 ng/ml DOX together with highest concentrations of indicated β-CATENIN inhibitors (IWR-1 50 µM and XAV939 40 µM). This reveals that increased Active β-CATENIN upon DUX4 induction is reduced by concomitant blockade of β-CATENIN activation, which does not affect DUX4 accumulation (quantification in Supplementary Figure S5F). N = 3 biological replicates, ANOVA, Tukey’s posthoc test. Ratio of Total/Active β-CATENIN is reported as fold change to DOX-treated (+) samples. **(E)** RT-qPCR analysis on iDUX4 myoblasts treated as in D, showing effect of DUX4 induction and inhibition of β-CATENIN signalling on expression of *LGR5* and the DUX4 target gene *PRAMEF1*. N = 3 wells, ANOVA, Holm-Sidak’s posthoc test. Relative expression as fold change to NI iDUX4 sample is reported. Graphs report mean ± SEM with statistical significance between specific samples indicated by a bar.

Our transcriptomic analysis retrieved the biological process WNT/β-CATENIN within genes up-regulated by DUX4 and down-regulated by DUX4c (Figures 3D,F). Western blot analysis of iDUX4 myoblasts induced with 62.5 or 125 ng/ml of DOX suggested DOX-dependent increase in active (non-phosphorylated) β-CATENIN (Supplementary Figures S4A, S5A), confirming that DUX4 perturbs the WNT/β-CATENIN pathway. Since activity by β-CATENIN is crucial for myoblast homeostasis and strongly relies on its cellular location (Krieghoff et al., 2006; Valenta et al., 2012; Suzuki et al., 2015; Agley et al., 2017), we assessed the effect of DUX4 on β-CATENIN localisation. Immunolabelling revealed that virtually all non-induced iDUX4 myoblasts displayed cytoplasmic β-CATENIN (Figure 4A; Supplementary Figure S4B). In contrast, DUX4 induction caused a robust accumulation of β-CATENIN in the nucleus (Figure 4A; Supplementary Figure S4B), also confirmed by concurrent nuclear immunoreactivity of the non-phosphorylated ‘active’ form of β-CATENIN (Figure 4B). Moreover, dose-dependent DOX treatment revealed that up-regulation of DUX4 positively correlates with increased accumulation of β-CATENIN, with lower doses (15.6, 31.3, or 62.5 ng/ml DOX) leading to nuclear localisation in approximately 12% of myoblasts, whereas higher concentrations (125 or 250 ng/ml DOX) further enhanced the percentage to 25%–30% (Figure 4A; Supplementary Figure S4C). Thus, DUX4 induces nuclear translocation of active β-CATENIN.

To assess whether β-CATENIN activation is required for DUX4 cytotoxicity, we evaluated the effect of β-CATENIN blockade on DUX4-induced apoptosis. iDUX4 myoblasts were cultured in growth medium and co-treated for 24 h with DOX and either IWR-1 or XAV939: β-CATENIN inhibitors that block Tankyrases-dependent β-CATENIN stabilisation (Supplementary Figure S5A). Importantly, inhibition of β-CATENIN with either IWR-1 or XAV939 caused little to no toxicity compared with the DMSO used to dissolve the inhibitors in non-induced iDUX4 myoblasts, using the Annexin V apoptosis assay (Supplementary Figures S5B–D). Strikingly, inhibition of β-CATENIN with either IWR-1 or XAV939 significantly reduced DUX4-induced apoptosis (Figure 4C; Supplementary Figure S5E). Both inhibitors efficiently blocked DUX4-induced β-CATENIN activation and expression of its target gene *LGR5* but did not alter DUX4 accumulation and expression of its target gene *PRAMEF1* compared with non-induced iDUX4 myoblasts (Figures 4D,E; Supplementary Figures S5F,G). We conclude that DUX4 toxicity requires active WNT/β-CATENIN signalling.

### DUX4c Reduces DUX4-Induced Nuclear Localisation β-CATENIN and Transcriptional Activity

**FIGURE 5:**
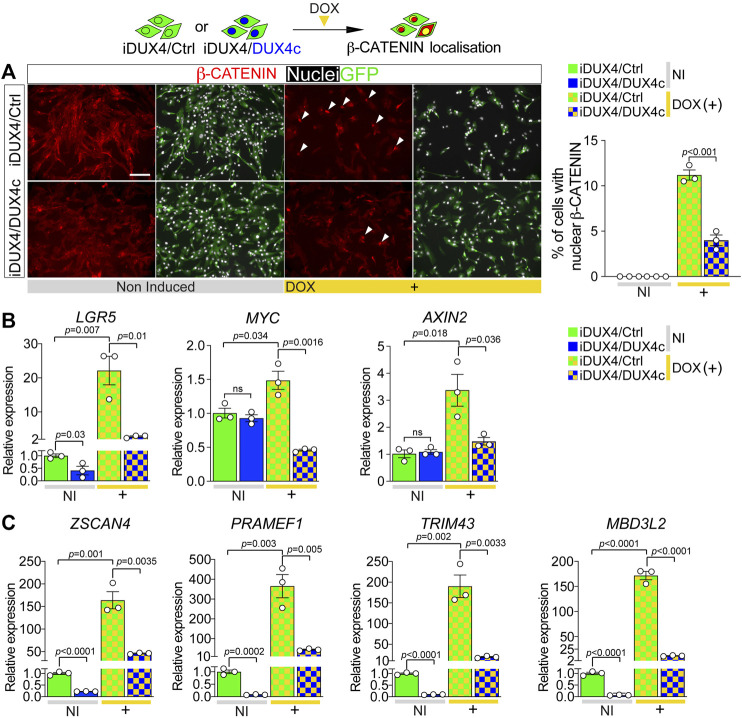
DUX4c reduces DUX4-induced β-CATENIN signalling and expression of DUX4 target genes. **(A)** Representative images of proliferating iDUX4/Ctrl and iDUX4/DUX4c myoblasts induced with 62.5 ng/ml DOX (+) and co-immunolabelled for β-CATENIN (red) and GFP (green), with nuclei counterstained with DAPI (white). Arrowheads indicate iDUX4/Ctrl myoblasts with nuclear β-CATENIN in DOX-treated samples. Scale bar equals 100 µm. Quantification of the percentage of iDUX4/Ctrl and iDUX4/DUX4c myoblasts with nuclear β-CATENIN when non-induced (NI) or after treatment with 62.5 ng/ml DOX (+). β-CATENIN nuclear localisation in DOX-induced iDUX4/Ctrl myoblasts is significantly higher than in DOX-induced iDUX4/DUX4c myoblasts. N = 3 biological replicates, ANOVA, Tukey’s posthoc test. **(B)** RT-qPCR analysis on iDUX4/Ctrl and iDUX4/DUX4c myoblasts when non-induced (NI) or after treatment with 62.5 ng/ml DOX (+) for 24 h showing up-regulation of the β-CATENIN target genes *LGR5, MYC,* and *AXIN2* upon DUX4 induction in iDUX4/Ctrl compared to non-induced control but not in DOX-induced iDUX4/DUX4c myoblasts. N = 3 biological replicates, ANOVA, unpaired two tailed t-test. **(C)** RT-qPCR analysis on iDUX4/Ctrl and iDUX4/DUX4c myoblasts treated as in B, showing the repressive effect of constitutive DUX4c expression on the DUX4 target genes *ZSCAN4*, *PRAMEF1*, *TRIM43*, and *MBD3L2* mRNA levels compared to iDUX4/Ctrl in both non-induced and DOX-induced iDUX4/DUX4c. N = 3 biological replicates, ANOVA, Tukey’s posthoc test. Relative expression as fold change to NI iDUX4/Ctrl sample is reported. Graphs report mean ± SEM with statistical significance between specific samples indicated by a bar.

We next assessed the effect of DUX4c on DUX4-induced β-CATENIN nuclear translocation and its activation of target genes. As per untransduced iDUX4 cells (Figures 4A,B), β-CATENIN was predominantly cytoplasmic in non-induced iDUX4/Ctrl or iDUX4/DUX4c myoblasts, suggesting that constitutive expression of DUX4c *per se* has no detectable effect on β-CATENIN localisation (Figure 5A). As expected, induction of DUX4 led to nuclear accumulation of β-CATENIN in iDUX4/Ctrl myoblasts (Figure 5A). In contrast, fewer iDUX4/DUX4c myoblasts had DUX4-induced nuclear β-CATENIN compared to control iDUX4/Ctrl myoblasts (Figure 5A). Thus, DUX4c suppresses DUX4-induced β-CATENIN accumulation in the nucleus.

β-CATENIN in the nucleus associates with transcription factors such as TCF/LEF to regulate expression of target genes (Valenta et al., 2012), so we assessed levels of the WNT/β-CATENIN early responder genes *LGR5*, *MYC*, and *AXIN2* (Figeac and Zammit, 2015). Consistent with unchanged levels of nuclear β-CATENIN, constitutive DUX4c expression had no effect on β-CATENIN target genes in non-induced iDUX4/DUX4c myoblasts, with *LGR5* expression actually being significantly reduced (Figure 5B). In line with augmented nuclear located β-CATENIN, expression of β-CATENIN target genes was significantly increased upon DUX4 induction in iDUX4/Ctrl myoblasts (Figure 5B). In contrast, concomitant DUX4c expression in DOX-induced iDUX4/DUX4c myoblasts attenuated the increase in these early responder genes, indicating that DUX4c reduces DUX4 toxicity by reversing β-CATENIN translocation to the nucleus and so blunting subsequent effects on WNT/β-CATENIN target genes.

### DUX4-Mediated WNT/β-CATENIN Activation Does Not Promote Myogenic Differentiation

Since WNT/β-CATENIN signalling is crucial for human myogenesis (Agley et al., 2017) and overexpression of DUX4 and/or DUX4c perturbs myogenic differentiation (Figure 2), we examined whether the DUX4-induced increase in nuclear β-CATENIN would enhance myogenic differentiation, thereby explaining reduced proliferation and onset of apoptosis. Up-regulation of either DUX4 or DUX4c severely hampered expression of *MYOD* and *MYF5,* arguing against promotion of the differentiation program (Supplementary Figure S6A). Concomitant expression of DUX4 and DUX4c in iDUX4/DUX4c myoblasts further suppressed *MYOD* and *MYF5* expression levels, suggesting a synergistic effect (Supplementary Figure S6A) and confirming some transcriptional target overlap.

Upon differentiating stimulus, myoblasts upregulate *CDKN1A* (P21) and exit the cell cycle (Zhang et al., 1999). However, the *CDKN1A* level decreased significantly upon 24 h of DUX4 induction, and was not rescued by concurrent DUX4c expression in iDUX4/DUX4c myoblasts, again arguing against DUX4-promoting initiation of myogenic differentiation. In contrast, DUX4 induction led to significant up-regulation of *MYOG*, but not of its target genes *MYMK* and *MYH2*, implying uncoordinated and premature signalling that does not lead to DUX4-induced terminal differentiation (Supplementary Figure S6A). Evaluation of publicly available transcriptomic data from DOX-induced iDUX4 myoblasts (GSE78158) (Choi et al., 2016) to assess expression of genes encoding myogenic regulatory factor (MRFs), myocyte enhancer factors 2 (MEF2s), and several sarcomeric components, confirmed no consistent trend towards a coordinated initiation of the myogenic differentiation program (Supplementary Figure S6B). We conclude that DUX4 leading to WNT/β-CATENIN activation/nuclear accumulation does not promote myogenic differentiation.

### DUX4c Suppresses the Expression of DUX4 Target Genes

DUX4 cytotoxicity is thought mediated through expression of its target genes (Choi et al., 2016; Lim et al., 2020; Banerji and Zammit, 2021); thus, we investigated the effects of DUX4c expression on the DUX4 transcriptome. Levels of known DUX4 target genes *ZSCAN4*, *PRAMEF1*, *TRIM43*, and *MBD3L2* were evaluated in iDUX4/Ctrl or iDUX4/DUX4c myoblasts. Non-induced iDUX4/DUX4c myoblasts have low but detectable levels of DUX4 target gene expression, likely due to ‘leaky’ basal DUX4 expression (Choi et al., 2016) (Figure 4E; Supplementary Figures S4A, S5F). Constitutive DUX4c expression significantly reduced even this low level DUX4 target gene expression (Figure 5C). DOX-induced DUX4 resulted in significant up-regulation of all four DUX4-target genes in iDUX4/Ctrl myoblasts by >150 fold, whereas DOX-induced iDUX4/DUX4c myoblasts exhibited significantly less up-regulation (Figure 5C). Thus, DUX4c can counteract activation of the DUX4 transcriptomic program along with its downstream signalling cascades.

### DUX4/DUX4c Antagonism Converges on FSHD Myoblast Viability via β-CATENIN

Since DUX4 up-regulation alters endogenous levels of *DUX4c*, we sought to examine whether FSHD myogenic cells, where DUX4 is expressed, also display reduced *DUX4c* expression.

**FIGURE 6:**
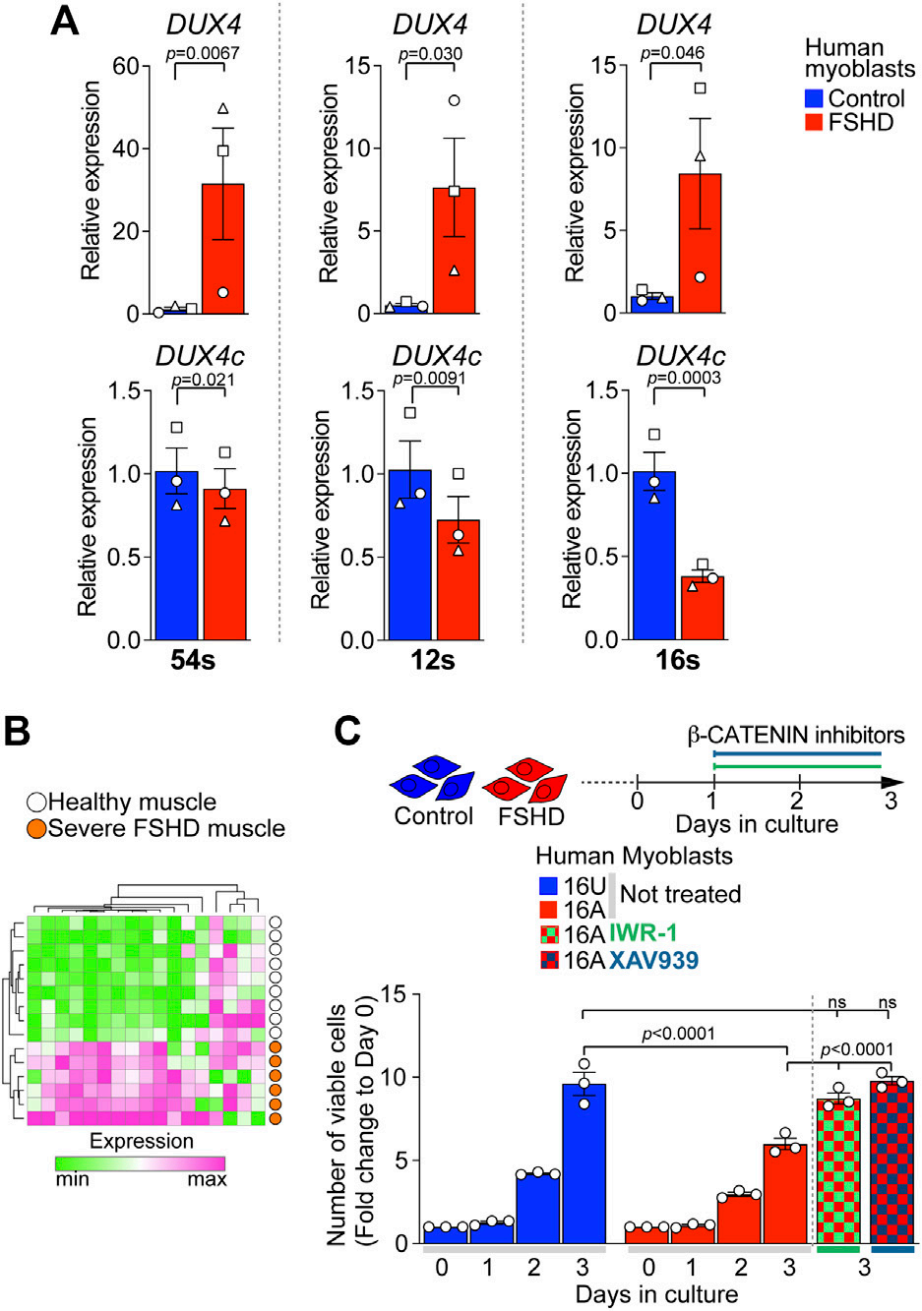
Reduced DUX4c expression in FSHD myoblasts and rescue of proliferation via β-CATENIN inhibition. **(A)** RT-qPCR analysis on the ‘54s’, ‘12s’, and ‘16s’ patient-derived immortalised myoblast lines showing higher expression of DUX4 and lower expression of DUX4c in all FSHD clones (54-12, 12A, and 16A) compared to unaffected matched control cells (54-6, 12U, and 16U) N = 3 biological replicates, paired t-test, symbols indicate independent biological replicates within same cell line. **(B)** 17/88 of genes differentially expressed in opposite directions by DUX4 and DUX4c relate to WNT/β-CATENIN signalling. A heatmap depicts that expression of this 17 gene subset separates human muscle biopsies of severe (Group 4) FSHD patients (*n* = 6, orange) from healthy controls (*n* = 9, white) from GSE115650 (Wang et al., 2019). **(C)** Cell viability assayed by counting number of cells over 3 days from plating indicates lower proliferation rate in 16A FSHD myoblasts compared to 16U controls. Notably, 48 h treatment with either IWR-1 (5 μM) or of XAV939 (4 μM) fully rescues 16A proliferation to the level observed in16U control cells. Graphs report mean ± SEM from representative experiments with statistical significance between samples indicated by a bar.

We evaluated the expression level of *DUX4* and *DUX4*c in three independent patient-derived immortalised FSHD myoblast lines. As expected, FSHD myoblasts (54-12, 12A, and 16A) displayed significantly higher levels of *DUX4* compared to their D4Z4 non-contracted control clone (54-6) or sibling-matched unaffected controls (12U and 16U) (Figure 6A). In contrast, *DUX4c* expression was significantly lower in all FSHD myoblasts compared to matched controls (Figure 6A), resembling the situation in DOX-induced iDUX4 myoblasts (Figure 3) and suggesting that reduced DUX4c levels may contribute to enhanced DUX4-induced toxicity.

WNT/β-CATENIN has previously been implicated in FSHD pathology and positively correlates with the regulation of CASP3-mediated apoptosis in FSHD muscle biopsies (Banerji et al., 2015). Interestingly, we found that 17/88 (19%) of our target genes differentially regulated by DUX4 and DUX4c (Figure 3) were annotated under GOs referring to WNT/β-CATENIN signalling (Supplementary Table S4), confirming that DUX4 and DUX4c converge on this pathway. Strikingly, the expression pattern of these 17 genes alone was sufficient to separate FSHD patients with high expression of four DUX4 target genes and severe pathology (Group 4 in (Wang et al., 2019)) from healthy muscle biopsies (Figure 6B) using GSE115650. This further strengthens our hypothesis that the DUX4:DUX4c ratio contributes to altered β-CATENIN signalling in FSHD muscles, and the likelihood that these 17 genes could be a biomarker for severe FSHD.

FSHD myoblasts have a reduced proliferation rate compared to matched unaffected lines (Moyle et al., 2016). Since DUX4 reduces cell viability via the activation of β-CATENIN target genes, which DUX4c can counteract, we tested the effects of inhibiting WNT/β-CATENIN signalling with IWR-1 or XAV939 on the viability of 16A FSHD myoblasts, which display the lowest expression of DUX4c among the tested lines (Figure 6A). FSHD 16A and control 16U myoblasts were plated at equal numbers and cultured in growth medium. 24 h after plating, myoblasts were treated with either IWR-1, XAV939 or vehicle for 2 days. Cell counting confirmed fewer FSHD 16A myoblasts compared to unaffected 16U cells (Figure 6C) when given vehicle alone. However, a 48 h blockade of β-CATENIN signalling with either IWR-1 or XAV939 fully rescued FSHD 16A myoblast numbers (Figure 6C). Importantly, neither IWR-1 nor XAV939 altered the number of 16U control myoblasts compared to vehicle alone (Supplementary Figure S6C). Thus, dysregulation of the β-CATENIN pathway due to DUX4 is modified by DUX4c molecular antagonism in FSHD pathomechanisms.

## Discussion

We report that DUX4 affects WNT/β-CATENIN signalling to cause deleterious effects in FSHD and that molecular antagonism between DUX4 and DUX4c moderates FSHD pathomechanisms through seven major findings. i) DUX4c robustly reduces expression of DUX4 target genes, while DUX4 induction decreases endogenous *DUX4c* expression in myoblasts. ii) DUX4 and DUX4c differentially regulate a subset of genes in opposite directions, defining a transcriptomic signature exploitable as a FSHD biomarker. iii) DUX4c rescues the anti-proliferative effects of DUX4 and attenuates DUX4-induced CASP3-mediated apoptosis. iv) DUX4 causes apoptosis through nuclear accumulation of β-CATENIN, along with the activation of the β-CATENIN-mediated signalling program. v) DUX4c blunts DUX4-mediated activation of β-CATENIN signalling. vi) A minimal transcriptomic signature that highlights DUX4/DUX4c convergence on the β-CATENIN signature efficiently separates severe FSHD from healthy control muscle biopsies. vii) β-CATENIN inhibition efficiently rescues proliferation of FSHD myoblasts.

Together, our observations suggest that antagonism between DUX4 and DUX4c contributes to moderating FSHD pathogenesis via balancing WNT/β-CATENIN signalling.

In human rhabdomyosarcoma cell lines, DUX4c is reported to contribute to cell viability (Ansseau et al., 2009) implying some pro-proliferative activity under pathological circumstances. We found that constitutive expression of DUX4c did not significantly alter the proliferation rate of wild-type human myoblasts. This suggests that the ability of DUX4c to rescue the DUX4-mediated suppression of proliferation is via interfering with DUX4 activity. Congruent with our data, DUX4c does not alter ATP production in murine myoblasts, in sharp contrast to DUX4 (Bosnakovski et al., 2008a), again arguing for DUX4/DUX4c molecular antagonism. DUX4c knockdown in human FSHD myoblasts further reduces proliferation, while DUX4c overexpression promotes it (Vanderplanck et al., 2018), suggesting that DUX4c actively buffers DUX4 toxicity in FSHD. Indeed, DUX4 up-regulation results in decreased DUX4c transcripts in iDUX4 myoblasts, and there is reduced DUX4c expression in three unrelated DUX4-expressing patient-derived FSHD myoblast lines compared to matched control cells. These observations suggest that DUX4 may actively inhibit DUX4c expression, thereby potentiating its effect on cell viability.

A D4Z4 proximally extended deletion (DPED) allele termed the “D4F104S1 genomic deletion”, which extends from the D4Z4 repeat array to include the *DUX4c* and *FRG2* loci, was reported in two FSHD families (Lemmers et al., 2003; Deak et al., 2007). This implies that DUX4c is not causative of FSHD pathology. However, one family with the DPED allele showed severe symptoms (Lemmers et al., 2003) suggesting that reduction in *DUX4c* function may worsen FSHD, consistent with our hypothesis that DUX4c attenuates DUX4 toxicity. Indeed, primary myoblasts from FSHD patients bearing the DPED allele have significantly higher levels of *DUX4* and of its target genes *TRIM43*, *MBD3L2*, and *ZSCAN4*, compared with FSHD samples containing the *DUX4c* locus. This suggests that lack of *DUX4c*, and/or *FRG2* further enhances DUX4 toxicity (Lemmers et al., 2021). However, *FRG2* is directly activated by DUX4 (Thijssen et al., 2014) and *FRG2* and *DUX4* expression positively correlate (Choi et al., 2016), so it is unlikely that *FRG2* deletion would enhance DUX4 transactivity. Strikingly, increased DUX4 activity was also shown in DPED patient-derived fibroblasts transdifferentiated into the myogenic lineage (Lemmers et al., 2021), further strengthening the hypothesis that DUX4c buffers DUX4 function in FSHD. DPED alleles were initially estimated to account for 2%–3% of FSHD cases, but are now estimated at 0.6% (Lemmers et al., 2021), so comparatively rare. It is now essential to assess whether the levels of *DUX4c* correlates with FSHD age of onset or disease severity.

Given the complete sequence homology between the two DNA-binding homeodomains of DUX4 and DUX4c, it is unsurprising that over 75% (268/356) of human gene orthologs differentially regulated by DUX4 and DUX4c in mouse are either up- or down-regulated by both DUX4 and DUX4c, although differences in the magnitude of change are likely due to the strong C-terminal transactivation domain of DUX4 (Knopp et al., 2016; Vanderplanck et al., 2018). The protective effect of DUX4c may in part arise from competitive inhibition with DUX4 on the remaining 25% (88/356) of human ortholog genes that display opposite expression trajectories upon DUX4 or DUX4c expression in mouse, further demonstrating the antagonism between DUX4 and DUX4c. While there are transcriptomes from DUX4 expression in human myoblasts, there are currently no such datasets after DUX4c expression in human myoblasts, so we cannot easily directly test the control of these 88 genes by DUX4 and DUX4c in human. Strikingly, this 88 gene-set also clusters severe FSHD from healthy muscle biopsies based on expression pattern.

We previously reported a positive correlation between CASPASE signalling and β-CATENIN in the FSHD interactome (Banerji et al., 2015), indicating interplay between the two molecular cascades. We confirmed that DUX4-mediated cell death relies on CASPASE signalling, since DUX4c protects myoblasts from DUX4 cytotoxicity, repressing Caspase3/7 activation and preventing subsequent apoptosis. Moreover, direct blockade of β-CATENIN function significantly attenuates DUX4-induced cell death, further strengthening the link between DUX4, β-CATENIN and apoptosis in FSHD muscle.

Uncontrolled activation of WNT/β-CATENIN signalling causes increased mitochondrial-derived reactive oxygen species (ROS) production and induces oxidative damage in murine muscle cells (Yoon et al., 2010). In turn, mitochondrial dysfunction and accumulation of mitochondrial ROS can elicit the onset of an apoptotic program via CASPASE signalling (Circu and Aw, 2010). We recently reported disturbed mitochondrial ROS metabolism upon DUX4 accumulation in iDUX4 myoblasts and in FSHD muscle cells (Heher et al., 2022). This, together with our findings here, suggests that DUX4-induced activation of WNT/β-CATENIN leads to cell toxicity by causing mitochondria dysfunction and downstream CASPASE activation in FSHD.

We found that a subset of 17 of the 88 (19%) genes differentially regulated upon DUX4 or DUX4c expression refer to WNT/β-CATENIN signalling. This 17-gene signature also efficiently separates severe FSHD patients from healthy controls, providing a minimal transcriptomic signature suitable for molecular profiling of severe FSHD patients. Importantly, previous transcriptomic analysis also highlighted an enrichment for altered mitochondrial pathways in the same dataset (Heher et al., 2022) and in FSHD muscle cells (Banerji et al., 2019), further arguing for a DUX4/β-CATENIN/mitochondrial functional network operating in FSHD pathogenesis.

Canonical WNT signalling is also key to both myoblast proliferation and differentiation (Murphy et al., 2014; Figeac and Zammit, 2015; Rudnicki and Williams, 2015), with β-CATENIN continuously shuttling between the nucleus and cytoplasm (Krieghoff et al., 2006; Agley et al., 2017). In the nucleus, β-CATENIN acts as a transcriptional activator or repressor, through TCF/LEF transcription factors (Valenta et al., 2012). WNT/β-CATENIN signalling is altered in FSHD muscle and DUX4 overexpression affects β-CATENIN signalling *in vitro* (Banerji et al., 2015). We show that DUX4 causes β-CATENIN to accumulate in the nucleus of proliferating human myoblasts, presumably by affecting translocation from cytoplasm to the nucleus, thereby affecting subsequent transcriptional regulation of target genes. This provides a molecular link between DUX4 toxicity and disrupted WNT/β-CATENIN signalling in FSHD pathogenesis, in line with observations in murine myoblasts (Banerji et al., 2015; Agley et al., 2017). Notably, DUX4 stabilises its own transcripts through repression of Non-sense Mediated Decay (NMD) machinery (Feng et al., 2015), so it will be interesting to determine if this also operates through WNT/β-CATENIN signalling.

It has been suggested that overexpression of DUX4c in FSHD myotubes may lead to some β-CATENIN accumulation, further confirming that DUX4 and DUX4c converge on this pathway during myogenesis. Both DUX4 and DUX4c may contribute to β-CATENIN regulation by interacting with the RNA helicases DDX5 and DDX17 (Ansseau et al., 2009; Janknecht, 2010). DDX5 directly interacts with β-CATENIN to enhance its function so it is possible that DUX4 and DUX4c may affect interactions with the β-CATENIN-DDX5/DDX17 complex. Moreover, inhibition of β-CATENIN degradation reduces DUX4 accumulation in differentiating FSHD human myoblasts preventing DUX4-dependent apoptosis (Block et al., 2013), further implying a regulatory feedback loop between DUX4 and the β-CATENIN network.

The effect of DUX4 on WNT/β-CATENIN signalling may have profound chronic effects on FSHD muscle homeostasis *in vivo*. β-CATENIN plays a central role in vertebrate myogenesis both in embryonic and in adult muscle (Schmidt et al., 2000; Agley et al., 2017). β-CATENIN activation is finely tuned to achieve the homeostatic balance between proliferation and differentiation in satellite cells (Brack et al., 2008; Murphy et al., 2014; Jones et al., 2015; Rudolf et al., 2016), with suppression of β-CATENIN function required for efficient regenerative myogenesis (Murphy et al., 2014). Thus, DUX4-induced alteration in WNT/β-CATENIN signalling would also contribute to inefficient muscle regeneration in FSHD (Banerji et al., 2020b). We recently defined FSHD as a secondary Satellite Cell-opathy (Ganassi et al., 2022; Ganassi and Zammit, 2022), given that DUX4 affects both satellite cell/myoblast and muscle fibre function, and suppression of PAX7 target genes (Banerji et al., 2017; Banerji and Zammit, 2021). Here, we found that DUX4-mediated activation of β-CATENIN signalling does not promote a coordinated ‘myogenic differentiation’ transcriptome. For example, DUX4 results in the up-regulation of *MYOG*, but down-regulation of *MYOD*, *MYF5*, and *CDKN1A. MYOG* expression fluctuates significantly in murine satellite cells, being high in quiescent cells, reduced rapidly during early activation before again being up-regulated to promote differentiation (Machado et al., 2017), in line with increased satellite cell activation in a zebrafish *Myog* null model (Ganassi et al., 2020; Ganassi et al., 2021). Such uncoordinated expression of myogenic genes would unbalance myogenic signalling and further stall adult myogenesis and muscle fibre regeneration.

Besides WNT/β-CATENIN activation, up-regulation of DUX4 results in accumulation of DUX4-target genes such as *TRIM43*, *PRAMEF1*, *MBD3L2*, *ZSCAN4,* and *KHDC1L* (Geng et al., 2012; Choi et al., 2016; Banerji et al., 2017; Banerji et al., 2020c; Lim et al., 2020; Banerji and Zammit, 2021). We found that the highest expression of such DUX4-targets is delayed about 9-10 h from the peak of *DUX4* mRNA. A temporal gap is expected between DOX addition to iDUX4 myoblasts, DUX4 expression, and consequent DUX4-mediated transcription. This resembles FSHD, where DUX4 mRNA/protein may quickly disappear, but accumulation of its target genes is protracted. While DUX4 is notoriously difficult to detect, its molecular signature is measurable in FSHD muscle biopsies (Yao et al., 2014; Rickard et al., 2015; Banerji et al., 2017; Wang et al., 2019; Banerji et al., 2020b) and has been suggested as a disease biomarker (Wang et al., 2019). In addition, chronic but sporadic/low DUX4 expression in mouse muscles recapitulates the molecular signature found in FSHD human biopsies (Bosnakovski et al., 2018; Bosnakovski et al., 2020).

Our study confirms that despite high sequence homology (but lack of a DUX4-like ‘activation domain’) (Choi et al., 2016; Bosnakovski et al., 2018), DUX4c does not induce accumulation of many DUX4 target genes, activating a largely distinct gene set (Bosnakovski et al., 2008a; Bosnakovski et al., 2008b; Knopp et al., 2016; Bosnakovski et al., 2018). Infact, constitutive expression of DUX4c in DUX4-induced iDUX4 myoblasts efficiently suppresses expression of *ZSCAN4*, *PRAMEF1*, *TRIM43*, and *MBD3L2*, some of which are up-regulated in patient-derived cells bearing the DPED allele, where one *DUX4c* locus is deleted (Lemmers et al., 2021). This indicates that DUX4c can inhibit DUX4 transactivity of specific target genes, possibly through interaction with chromatin modifiers or transcriptional repressors (Bosnakovski et al., 2018; Vanderplanck et al., 2018). This strengthens the hypothesis that despite conserved functions on some target genes, DUX4 and DUX4c compete to inhibit the other’s effects in FSHD. Indeed, we show that DUX4 represses *DUX4c* transcripts, while stabilizing its own mRNA (Feng et al., 2015). Finally, the inverse correlation between *DUX4* and *DUX4c* levels in FSHD myoblasts further supports reciprocal inhibition.

It may be that DUX4 levels and/or expression duration in an induced iDUX4 myoblast is higher/longer than in the muscle cell of an FSHD patient, yet DUX4c counteracts both high and residual ‘leaky’ expression of DUX4-target genes. A DUX4c re-expression strategy may be even more effective in FSHD muscle cells, where DUX4 is already difficult to even detect (e.g., (Vanderplanck et al., 2018)), but the ‘DUX4-signature’ is assayable, and in use to measure therapy effectiveness (ReDUX4 trial; NCT04003974). A 4-target gene DUX4-signature correlates with muscle disease severity (Wang et al., 2019), although DUX4 target gene biomarkers do not accord with disease progression (Banerji, 2020). Although our data confirm that constitutive expression of DUX4c may have a detrimental effect on terminal myogenic differentiation (Knopp et al., 2016), the therapeutic potential of DUX4c re-expression in FSHD muscle warrants further investigation. We previously developed a suicide-therapy approach for rhabdomyosarcoma in which the promoter region of *MYOG* (highly expressed in rhabdomyosarcoma) was modified to enhance rhabdomyosarcoma-specificity. Using this modified *MYOG* promoter to drive expression of the *HSV-TK* suicide gene resulted in specific targeting of tumour cells both *in vitro* and *in vivo* (Pruller et al., 2021). Such a system, for example using DUX4-binding DNA element(s) to express *DUX4c* specifically in FSHD cells, could counter DUX4 effects.

Together, our results highlight alternative potential interventions to suppress DUX4 toxicity, rather than attempting to further reduce levels of an already difficult-to-detect protein. DUX4 expression and its signalling cascade appear to be the root cause of FSHD, but the detrimental effects of the DUX4-driven molecular cascade extend beyond muscle. For example, immortalized FSHD lymphoblastoid cells display robust DUX4 expression and a DUX4-signature comparable to muscle, indicating an immune contribution in FSHD pathogenesis (Jones et al., 2017; Banerji et al., 2020c). In line, our GO analysis shows the biological process ‘Immune response’ as significantly enriched in the subset of genes up-regulated by DUX4 and down-regulated by DUX4c, suggesting that DUX4/DUX4c antagonism may take place in FSHD lymphoblasts in parallel to that we report in FSHD muscles/myoblasts. Moreover, a chromosomal translocation involving DUX4 homeodomains drives pathogenesis of acute lymphoblastic leukaemia (Yasuda et al., 2016). Since DUX4c can attenuate specific effects of DUX4, such as overactivation of the WNT/β-CATENIN, its tailored expression may be developed as a complementary route to therapy that could potentially benefit not only FSHD but also other DUX4-mediated conditions.

In summary, we found that DUX4 operates in part through a WNT/β-CATENIN pathomechanism to reduce myoblast proliferation and cause apoptosis. Molecular antagonism between DUX4 and DUX4c indicates that DUX4c is a genetic modifier of FSHD pathology.

## Materials and Methods

### Retroviral Expression Constructs

Human DUX4c cDNA was cloned into a modified *pMSCV-puro* vector (Clontech), in which the puromycin resistance gene has been replaced with an internal ribosomal entry site (IRES) preceding the coding sequence for enhanced green fluorescent protein (eGFP) to obtain *pMSCV-IRES-eGFP* (pMIG), as previously described (Banerji et al., 2015). For production of retroviral particles *pMSCV-DUX4c-IRES-eGFP* (RV_DUX4c-IRES-eGFP) or the empty control *pMSCV-IRES-eGFP* (RV_-IRES-eGFP) constructs were transfected in HEK293T cells, as previously described (Knopp et al., 2013; Knopp et al., 2016).

### Cell Culture

LHCN-M2 Myoblasts were previously engineered to express DUX4 under DOX control to generate LHCN-M2-iDUX (iDUX4) myoblasts (Choi et al., 2016). Retroviral transduction of iDUX4 myoblasts with RV_DUX4c-IRES-eGFP or RV_-IRES-eGFP was performed as previously described (Knopp et al., 2013). Transduced proliferating iDUX4 myoblasts were FACS for GFP positivity to obtain stable cell lines. Generated iDUX4/DUX4c, iDUX4/Ctrl, and non-transduced iDUX4 myoblasts were routinely maintained under selection with Puromycin (Sigma Aldrich), as previously described (Choi et al., 2016). iDUX4 myoblasts were grown in complete proliferation medium: Skeletal Muscle Cell Growth medium (PromoCell) supplemented with 20% heat-inactivated foetal bovine serum (FBS; Thermo Scientific), 50 μg/ml Gentamycin (Life Technologies), and 1 unit of the SupplementMix (PromoCell) and passaged at ∼70% confluency to maintain in ‘proliferation’. Myoblasts were plated at a density of 5 × 10^3^ cells/well in flat-bottomed 96-well plates for immunolabelling and at 1 × 10^6^ cells/well in 6-well plates for RT-qPCR. To induce DUX4 expression, cells were treated with varying concentrations of DOX (Clontech) for the indicated period of time. For proliferation assays, iDUX4 myoblasts in proliferation medium were pulsed with 10 mM EdU (Invitrogen) for 2 h immediately prior to fixation. Incorporated EdU was detected using the click-iT EdU AlexaFluor Kit (Invitrogen) according to the manufacturer’s instructions.

The three immortalised FSHD patient-derived cellular models were the isogenic ‘54’ series derived from the biceps of a male mosaic FSHD1 patient (Krom et al., 2012), where 54-6 (13 D4Z4 repeats) is the uncontracted control clone and 54-12 (3 D4Z4 repeats) the contracted FSHD clone. The ‘16s’ and ‘12s’ are immortalised models derived from biceps muscle (Homma et al., 2012) where 16A and 12A are the D4Z4-contracted FSHD lines and 16U and 12U are the uncontracted control lines from a first-degree relative. These FSHD cellular models were cultured as per the iDUX4 myoblasts.

β-CATENIN inhibitors were selected on the basis of reported effectiveness. IWR-1 and XAV939 (Generon) inhibit Tankyrases, thereby promoting degradation of β-CATENIN (Huang et al., 2009; Abraham, 2016). For assaying apoptosis, individual β-CATENIN inhibitors were added together with DOX at the time of induction on iDUX4 myoblasts. Drugs were diluted in DMSO and used at indicated final concentrations. The same volume of vehicle DMSO was used for controls.

### Immunolabelling and Imaging

For immunolabelling, iDUX4 myoblasts were fixed in 4% paraformaldehyde/PBS for 10 min, washed in 3 X PBS for 5 min and permeabilised for 5 min with 0.5% triton X100/PBS. Subsequently, cells were blocked for 1 h using 5% goat serum/PBS (blocking buffer). Primary antibodies were added in PBS and incubated overnight at 4°C. Primary antibodies were: rabbit polyclonal anti-Cleaved-CASPASE3 (Cell Signalling; 9661S; 1:400), mouse monoclonal anti-total β-CATENIN (BD; 610154; 1:200), rabbit monoclonal anti-Non phospho(Active)β-CATENIN (1:1000, 8814, Cell Signaling), chicken polyclonal anti-GFP (Abcam; ab13970; 1:1000), and mouse monoclonal anti-DUX4 (Millipore; 9A12; 1:1000). DUX4 antibody 9A12 recognises a region common to both DUX4 and DUX4c, so was also used to detect DUX4c expression as previously described (Bosnakovski et al., 2008a). Cells were then washed in 3 x PBS for 5 min, secondary antibodies added in blocking buffer and incubated for 1 h at room temperature. Secondary antibodies were: AlexaFluor 594 goat anti-mouse (Invitrogen; A11005; 1:1000) and Alexa Fluor 488 goat anti-chicken IgY (H + L) (Invitrogen; A11039; 1:1000). Nuclei were counterstained with 0.3 μM DAPI in PBS for 10 min and mounted in PBS. Cells were imaged using a classic Zeiss Axiovert 200 M epifluorescence microscope with a Zeiss AxioCam HRm and AxioVision 4.4 software (Zeiss, Jena, Germany).

### Western Blot

Western blot was performed as described previously (Ganassi et al., 2016; Mediani et al., 2020). Briefly, iDUX4 myoblasts were lysed for 30 min on ice using RIPA lysis buffer supplemented with 2 mM PMSF, 1 mM sodium orthovanadate, and protease inhibitor cocktail (PIC) (RIPA Lysis Buffer System, Santa Cruz) followed by sonication to obtain total protein lysate. Equal volumes of cell lysates were separated by SDS-PAGE and transferred onto nitrocellulose membrane (Amersham). After transfer, the membrane was blocked for 1 hour at room temperature in 5% skimmed milk in TBST buffer (25 mM TrisHCl, 137 mM NaCl, 0.1% Tween 20, pH 7.5) and probed with different antibodies. Incubation with primary antibody was performed overnight at 4°C followed by appropriate secondary HRP-conjugated antibodies (anti-rabbit IgG or anti-mouse IgG, 1:5000, Amersham) for 1 hour at room temperature. Enhanced chemiluminescence (ECL Plus, Amersham) was used for the detection of protein bands. The following primary antibodies were used: mouse monoclonal anti-total β-CATENIN (1:1000, L87A12, Cell Signaling), rabbit polyclonal anti-Non phospo(Active)β-CATENIN (1:1000, 8814, Cell Signaling), rabbit polyclonal anti-β-ACTIN (1:1000, 4970, Sigma); mouse monoclonal anti-DUX4 (1:1000, NBP1-49552, Novus Biological). Amersham ECL Prime Western blotting detection reagent was used for HRP detection (GE Healthcare). Signal detection was performed using ChemiDoc™ Imaging System (Bio-rad) and analysed using ImageJ (NIH, www.Fiji.sc). Uncropped scans of Western blots shown in this work are reported in Supplementary Figure S7.

### Cell Viability, Cell Death, and Caspase Activity Assays

For viability assay, iDUX4 myoblasts were plated at a density of 5 × 10^3^ cells/well in flat-bottomed 96-well plates. After 16 h, the medium was replaced with a 1:1 solution containing 2X RealTime-Glo MT Cell Viability reagent mixture (Promega) supplemented with DOX at the desired final concentration in a fresh proliferation medium following the manufacturer’s instructions. Luciferase signal was measured at 16, 24, 40, and 48 h of DOX treatment and displayed as fold change to the 16 h normalised for background.

For apoptosis/necrosis assays, cells were plated at a density of 1 × 10^4^ cells/well in flat-bottomed 96-well plates. After 16 h, the medium was replaced with a solution containing 1X RealTime-Glo Annexin V Apoptosis and Necrosis assay reagent mixture (Promega) supplemented with DOX, and with or without β-CATENIN inhibitors or DMSO vehicle, at the indicated final concentration in fresh proliferation medium following the supplier’s instructions. Apoptosis and necrosis were measured after 16 and 24 h, or as indicated, of DOX treatment and considered detectable when the signal increased over background levels.

For Caspase activity assay, cells were plated at a density of 1 × 10^4^ cells/well in flat-bottomed 96-well plates. After 16 h, the medium was replaced with a solution containing 1X Caspase-Glo3/7 Assay System reagent mixture (Promega) supplemented with DOX at the indicated final concentration in fresh proliferation medium following the supplier’s instructions. Activity of Caspase was measured at the indicated time points of DOX treatment and considered detectable when the signal increased over background levels.

For growth curve and cell count, 16A and 16U myoblasts were plated at a density of 2 × 10^5^ cells/well in 24-well plates. For β-CATENIN inhibition after 24 h, the medium was replaced with fresh medium containing either 5 μM of IWR-1 or 4 μM of XAV939. At indicated time points, cells were harvested, and the number of viable cells was counted with a haemocytometer upon Trypan Blue staining.

### RNA Extraction, Reverse Transcription, and Quantitative PCR

Total RNA was extracted using the RNeasy kit (Qiagen) and quantified using a NanoDrop before being retrotranscribed with QuantiTect Reverse Transcription Kit (Qiagen) or SuperScript First-Strand Synthesis System (Thermo Scientific). RT-qPCR was carried out using MasterMix solution (Qiagen) or Takyon Low ROX SYBR 2X MasterMix blue dTTP (Takyon) as per the manufacturer’s instructions on a ViiA7 thermal cycler (Applied Biosystems). RT-qPCR analyses were performed as previously described (Ganassi et al., 2014; Ganassi et al., 2018; Ganassi et al., 2020; Ortuste Quiroga et al., 2022). Ct values of genes analyzed were normalized to the Ct values of the housekeeping genes *TBP* or *RPLP0* and fold changes were calculated using the ΔΔCt method (Livak and Schmittgen, 2001). Results are presented as mean value ±SEM of fold changes from independent experiments as indicated. Primers were purchased from Sigma Aldrich and sequences are reported in Supplementary Table S1.

### Data Analysis

Three images were taken per well at ×10 magnification for each replicate and counted manually using ImageJ. For immunolabelling, data are presented as the mean proportion of total DAPI-positive cells ±SEM, N = 3 biological replicates. For RT-qPCR, data were presented as average relative expression ±SEM, N = 3. Data presented as mean ± SEM from N = 3/4 independently treated wells, considered biological independent replicates, from a representative experiment(s). Statistical significance was calculated in GraphPad Prism using unpaired t-test or one-way ANOVA followed by Tukey’s post hoc or Holm-Sidak tests. Paired t-test was used to account for DUX4/DUX4c variation across FSHD myoblast samples.

Alignment of human DUX4 (NP_001280727.1) and DUX4c (Q6RFH8.1) protein sequences, retrieved from Ansseau et al. (2009), was performed using Clustal Omega (ebi.ac.uk/Tool/msa/clustalo (Madeira et al., 2019)) with default parameters. Identity between DUX4 and DUX4c homeodomains, or over entire protein sequences, was confirmed using the NCBI conserved-domain-search tool (ncbi.nlm.nih.gov/Structure) and validated using the Expasy SIM alignment tool (web.expasy.org/sim) both with default parameters.

Transcriptomic analysis on differential expressed genes upon human DUX4 or DUX4c overexpression in murine satellite cell-derived myoblasts (Banerji et al., 2015; Knopp et al., 2016) (GSE77100) was performed using GEO2R (https://www.ncbi.nlm.nih.gov/geo/geo2r/) with default settings (Figure 2A). Differential expression analysis was designed by defining three groups 1) RV_ctrl (empty pMIG construct) vs. RV_DUX4c (in pMIG), R_-ctrl vs. RV_DUX4 (in pMIG) and RV_DUX4 vs. RV_DUX4c. The resulting 394 differentially expressed murine probes (microarray) were converted using g:Orth (https://biit.cs.ut.ee/gprofiler/orth) and resulted in 356 human genes (Figure 3C). The list was sorted to identify genes with opposite expression trends upon DUX4 or DUX4c accumulation, resulting in 88 genes (47 up-regulated by DUX4c and down-regulated in DUX4; 41 down-regulated by DUX4c and up-regulated by DUX4) (Supplementary Table S2). Gene Ontological (GO) analysis was performed on the two sets separately (47 genes and 41 genes) using Metascape (https://metascape.org, (Zhou et al., 2019)) with default settings. Gene Ontologies (GO terms) referring to Biological Processes and with FDR≤0.01 were visualised using Cytoscape (cytoscape.org; v3.8.2; (Shannon et al., 2003; Badodi et al., 2021; Badodi and Marino, 2022)) and clustered in Main Biological Processes. Layout parameters were optimised for presentation. Bubbles are coloured based on False Discovery Rate (FDR) values and size is proportional to number of genes within specific GO terms, grey lines represent genes shared across different GOs.

Gene counts of human severe FSHD (6 samples) and healthy muscle biopsies (9 samples) were derived from Wang et al. (2019) (GSE115650) and normalised to Count per Million (CPM). The selected FSHD sample group (6) refers to patients with highest DUX4 target gene expression and most severe muscle pathology (‘group 4’) as previously described (Wang et al., 2019). To assay onset of ‘terminal differentiation’ genes upon DOX-induced DUX4 up-regulation in iDUX4 myoblasts, expression values were retrieved from GSE78158 (Choi et al., 2016) as FPKM (Fragments Per Kilobase of transcript per Million reads) and presented as a heatmap. Genes were classified into ‘MRFs’ (Myogenic Regulatory Factors), ‘MEF2s’ (Myocyte Enhancer Factor 2s) (Magli et al., 2010; Badodi et al., 2015; Baruffaldi et al., 2017), or ‘Sarcomere’ according to the current literature. Heatmaps were created using Morpheus (https://software.broadinstitute.org/morpheus) and applying ‘One plus Log2’ and ‘Zscore’ adjustments to CPM values of selected genes. Morpheus hierarchical clustering was applied blindly to assess the goodness of the 88 (Figure 3D; Supplementary Table S3) or 17 genes (Figure 6B; Supplementary Table S4) to cluster separately FSHD samples from healthy controls based on gene expression pattern. The 17 genes used in Figure 6B were retrieved from the list of RV-DUX4 vs. RV-DUX4c differentially expressed genes involved in WNT/β-CATENIN signalling and collecting all genes annotated under GO terms containing the word ‘WNT’ or ‘β-CATENIN’ (Supplementary Table S4 for details) following GO analysis using Metascape on all 88 genes taken together.

## Data Availability

The datasets presented in this study can be found in online repositories. The names of the repository/repositories and accession number(s) can be found at: https://www.ncbi.nlm.nih.gov/genbank/, GSE77100; https://www.ncbi.nlm.nih.gov/genbank/, GSE115650; https://www.ncbi.nlm.nih.gov/genbank/, GSE78158 and are described in related publications [GSE77100; (Banerji et al., 2015; Knopp et al., 2016), GSE115650 (Wang et al., 2019) and GSE78158 (Choi et al., 2016)].

## Supplementary Material

Supplementary Figure S1 DUX4 rapidly induces expression of its target genes in human myoblasts and reduces cell viability. **(A)** Representative images of proliferating iDUX4 human myoblasts induced with 125 ng/ml Doxycyline (DOX) for 24 h and immunolabelled for DUX4 (red), with nuclei counterstained with DAPI (blue). Arrowhead indicates a nucleus containing DUX4 in the DOX-induced sample. Scale bar represents 100 µm. **(B)** Absolute number of DUX4 containing myoblasts per Field Of View (FOV) showing significant increase with any concentration of DOX used for 24 h. Notably, 62.5 ng/ml of DOX induced the highest number of DUX4^+ve^ nuclei. N = 3 biological replicates, ANOVA, Tukey’s posthoc test. **(C)** Proportion of DUX4^+ve^ myoblasts shows a positive correlation (magenta dotted line) between DOX dosage and DUX4 accumulation N = 3 wells, ANOVA, Tukey’s posthoc test. **(D)** Total (DAPI-stained) myoblasts per Field Of View (FOV) showing significant reduction at ≥62.5 ng/ml of DOX for 24 h. N = 3 biological replicates, ANOVA, Tukey’s posthoc test. **(E)** RT-qPCR analysis showing up-regulation of DUX4 mRNA followed by increased levels of DUX4-target genes using assay points 7, 16 and 24 h after 250 ng/ml DOX treatment on iDUX4 myoblasts. Comparison of relative expression revealed that high levels of DUX4 target genes occurred 9–10 h after the *DUX4* peak. N = 3 biological replicates, ANOVA, Tukey’s posthoc test. Relative expression as fold change to NI sample is reported. **(F)** Cellular viability at indicated time points following DOX treatment shows persistent reduction at indicated DOX concentrations. Coloured *p* values indicate significant difference among non-induced and DOX-treated samples (grey) and among timepoints within treated samples (magenta). N = 3 biological replicates, ANOVA, Tukey’s posthoc test. Measurement of Area Under Curve (coloured area underneath dashed grey, yellow or brown lines) to compare overall viability dynamics indicates significant reduction in viability upon DUX4 induction. Tukey’s posthoc test. Notably, treatment with higher dose of DOX (250 ng/ml) does not significantly reduce viability compared to the lower dose of 62.5 ng/ml. **(G)** Representative images of proliferating human iDUX4 myoblasts induced with 250 ng/ml of DOX for 24 h showing decrease in EdU (red) incorporation and morphological changes. All nuclei are counterstained with DAPI (white). Scale bar represents 100 µm. Proportion of EdU^+ve^ myoblasts treated with increasing DOX doses shows the inverse correlation between DUX4 accumulation and decrease in proliferation (magenta dotted line). N = 3 biological replicates, ANOVA, Tukey’s posthoc test. Statistical significance of DUX4-EdU incorporation correlation is shown in magenta. **(H)** Representative images of proliferating iDUX4 myoblasts induced with 62.5 ng/ml DOX showing signs of apoptosis (rounded cells) compared to non-induced controls. Magenta squares highlight ×6 magnified cells (arrowheads). Scale bar represents 100 and 50 µm for 6x zoom. Quantification of apoptosis and necrosis, after 24 h of DOX treatment at indicated concentrations, showing significant induction compared to non-induced control cells. Notably, 250 ng/ml DOX led to stronger induction of apoptosis compared to 62.5 ng/ml whereas necrosis was similarly triggered. N = 4 biological replicates, ANOVA, Tukey’s posthoc test. RLU; Relative Luciferase Units or RFU; Relative Fluorescent Units, reported as fold change to NI sample. Graphs report mean ± SEM from representative experiments. Statistical significance between samples indicated by bars. ns means not significant.

Supplementary Figure S2 Generation of human iDUX4 myoblasts constitutively expressing DUX4c. Schematic of retroviral infection (RV) on iDUX4 myoblasts with either the retroviral backbone encoding GFP (RV_-IRES-eGFP to obtain iDUX4/Ctrl) or encoding for both DUX4c and eGFP (RV_DUX4c-IRES-eGFP to obtain iDUX4/DUX4c) and selected via FACS to obtain stable constitutively expressing cell lines. **(A)** Representative images of uninduced proliferating iDUX4/Ctrl or iDUX4/DUX4c human myoblasts, co-immunolabelled for DUX4/DUX4c (red) and GFP (green). As the myoblasts were uninduced, immunolabelling mainly indicates DUX4c. **(B)** RT-qPCR analysis on stable uninduced iDUX4 myoblasts showing robust up-regulation of *DUX4c* mRNA in iDUX4/DUX4c myoblasts compared to iDUX4/Ctrl. N = 3 biological replicates, unpaired t-test. Relative expression as fold change to iDUX4/Ctrl sample is reported. Graphs report mean ± SEM from representative experiments with statistical significance between samples indicated a bar.

Supplementary Figure S3 Alignment of DUX4 and DUX4c protein sequences highlights identical homeodomains. **(A)** Schematic of DUX4 and DUX4c proteins showing the two homeobox domains and the C-terminal truncation (dashed red lines) in DUX4c compared to DUX4. Coloured dashed lines/rectangles highlight sequence identity within the selected protein regions. **(B)** Amino Acid sequence alignment shows high similarity between DUX4 and DUX4c over most of the protein sequences. Amino acid residues comprising Homeobox 1 and 2 are 100% identical and colour-coded as in A.

Supplementary Figure S4 DUX4 induces nuclear localisation of β-CATENIN and its activation. **(A)** Representative Western analysis on proliferating non-induced (NI) iDUX4 myoblasts and DOX-induced for 24 h showing increased Active β-CATENIN in parallel with DUX4 accumulation, after treatment with either 62.5 (+) or 125 ng/ml (++). N = 3 biological replicates, ANOVA, Tukey’s posthoc test. Ratio of Total/Active β-CATENIN and DUX4/ β-ACTIN are reported as fold change to NI samples. **(B)** Representative images of proliferating non-induced iDUX4 myoblasts and those induced with 125 ng/ml DOX (++) and immunolabelled for β-CATENIN (red) with nuclei counterstained with DAPI (blue). Arrowheads indicate β-CATENIN localisation. Scale bar represents 100 µm. **(C)** Quantification of iDUX4/Ctrl and iDUX4/DUX4c myoblasts with nuclear localized β-CATENIN nuclei (mean ± SEM) after treatment with increasing DOX doses, showing positive correlation between DUX4 accumulation and β-CATENIN nuclear translocation (magenta dotted line). N = 3 biological replicates, ANOVA, Tukey’s posthoc test with statistical significance between samples indicated with a bar. Statistical significance of the correlation between DUX4 and nuclear localisation of β-CATENIN is shown in magenta.

Supplementary Figure S5 Effect of β-CATENIN inhibitors on iDUX4 myoblasts. **(A)** Schematic of β-CATENIN inhibitors targeting the WNT/β-CATENIN signalling cascade, with IWR-1 and XAV939 blocking Tankyrases (TNKs) inhibition on β-CATENIN destruction complex. **(B)** Representative bright field images of proliferating human iDUX4 myoblasts treated for 24 h with IWR-1 (50 µM), XAV939 (40 µM) or vehicle DMSO showing little/no effect on cell viability. Scale bar equals 100 µm. **(C)** Quantification of apoptosis (Annexin V Luminescence) in iDUX4 myoblasts upon treatment with β-CATENIN inhibitors for 24 h shows no significant effect compared to vehicle treated control. RLU; Relative Luciferase Units reported as fold change to DMSO (vehicle) alone treatment. N = 4 biological replicates, ANOVA, Tukey’s posthoc test. **(D)** Quantification of apoptosis on iDUX4 myoblasts upon DOX-induced DUX4 accumulation for 24 h display DUX4-mediated toxicity when comparing untreated or DMSO iDUX4 myoblasts. RLU; Relative Luciferase Units reported as fold change to non-induced (NI) iDUX4. N = 4 biological replicates, ANOVA, unpaired two-tailed t-test. **(E)** Representative bright field images of proliferating human iDUX4 myoblasts co-treated for 24 h with DOX to induce DUX4 expression and either IWR-1 (50 µM) or XAV939 (40 µM) show reduced cell death in samples co-treated with β-CATENIN inhibitors. Scale bar equals 100 µm. **(F)** Representative Western analysis and quantification on proliferating non-induced (NI) and DOX-induced iDUX4 myoblasts induced with 62.5 ng/ml DOX (yellow line) showing unaltered DUX4 accumulation upon co-treatment with DOX and indicated β-CATENIN inhibitors compared to NI sample. N = 3 biological replicates, ANOVA, Tukey’s posthoc test. **(G)** Representative images of proliferating iDUX4 myoblasts treated as in E and immunolabelled for active β-CATENIN (green), DUX4 (red) with nuclei counterstained with DAPI (white) showing the effect of IWR-1 and XAV939 on β-CATENIN and DUX4. Scale bar represents 100 µm. Graphs report mean ± SEM from representative experiments with statistical significance between samples indicated by a bar.

Supplementary Figure S6 DUX4 accumulation dysregulates the myogenic differentiation transcriptome. **(A)** RT-qPCR analysis on iDUX4/Ctrl and iDUX4/DUX4c myoblasts when non-induced (NI) or after treatment with 62.5 ng/ml DOX (+) for 24 h showing alterations of *CDKN1A, MYOD, MYF5, MYOG, MYMK* or *MYH2* expression*.* N = 3 biological replicates, ANOVA, Tukey’s posthoc test. **(B)** Heatmap depicting expression of multiple genes involved in myogenic differentiation upon 6 h of 250 ng/ml DOX treatment (DOX) of iDUX4 myoblasts compared to non-induced cells (NI) retrieved from GSE78158 (Choi et al., 2016). Dots indicate the technical replicates of GSE78158, in bold genes assayed by RT-qPCR in A. **(C)** Cell viability assayed by counting number of cells over 3 days from plating indicates 48 h treatment with either IWR-1 (5 μM) or of XAV939 (4 μM) does not affect proliferation of 16U control cells. Graphs report mean ± SEM from representative experiments with statistical significance between samples indicated by a bar.

Supplementary Figure S7 Uncropped Western blots. **(A)** Uncropped Western blots with indicated molecular weights, antibodies and exposures used to generate the representative sections shown in Supplementary Figure 4A (pink dashed selections). **(B)** Uncropped Western blot with indicated molecular weights, antibodies and exposures used to generate the representative sections shown in Figure 4D (pink dashed selections).

Supplementary Table S1 List of primers used in this study.

Supplementary Table S2 List of genes deregulated upon DUX4 or DUX4c overexpression in murine myogenic cells shown in Figure 3.

Supplementary Table S3 Raw Data as Counts per Million (CPM) and data used by Morpheus to generate the heatmap of the 88 genes used for hierarchical clustering analysis presented in Figure 3.

Supplementary Table S4 List of 17 genes annotated under GOs referring to GOs referring to WNT/β-CATENIN signalling and presented in the heatmap in Figure 6B.
