# Supplementary figures and images for: Antagonism Between DUX4 and DUX4c Highlights a Pathomechanism Operating Through β-Catenin in Facioscapulohumeral Muscular Dystrophy
Source: Front Cell Dev Biol. 2022 Sep 7;10:802573. doi: 10.3389/fcell.2022.802573 (PMC9490378; doi:10.3389/fcell.2022.802573)

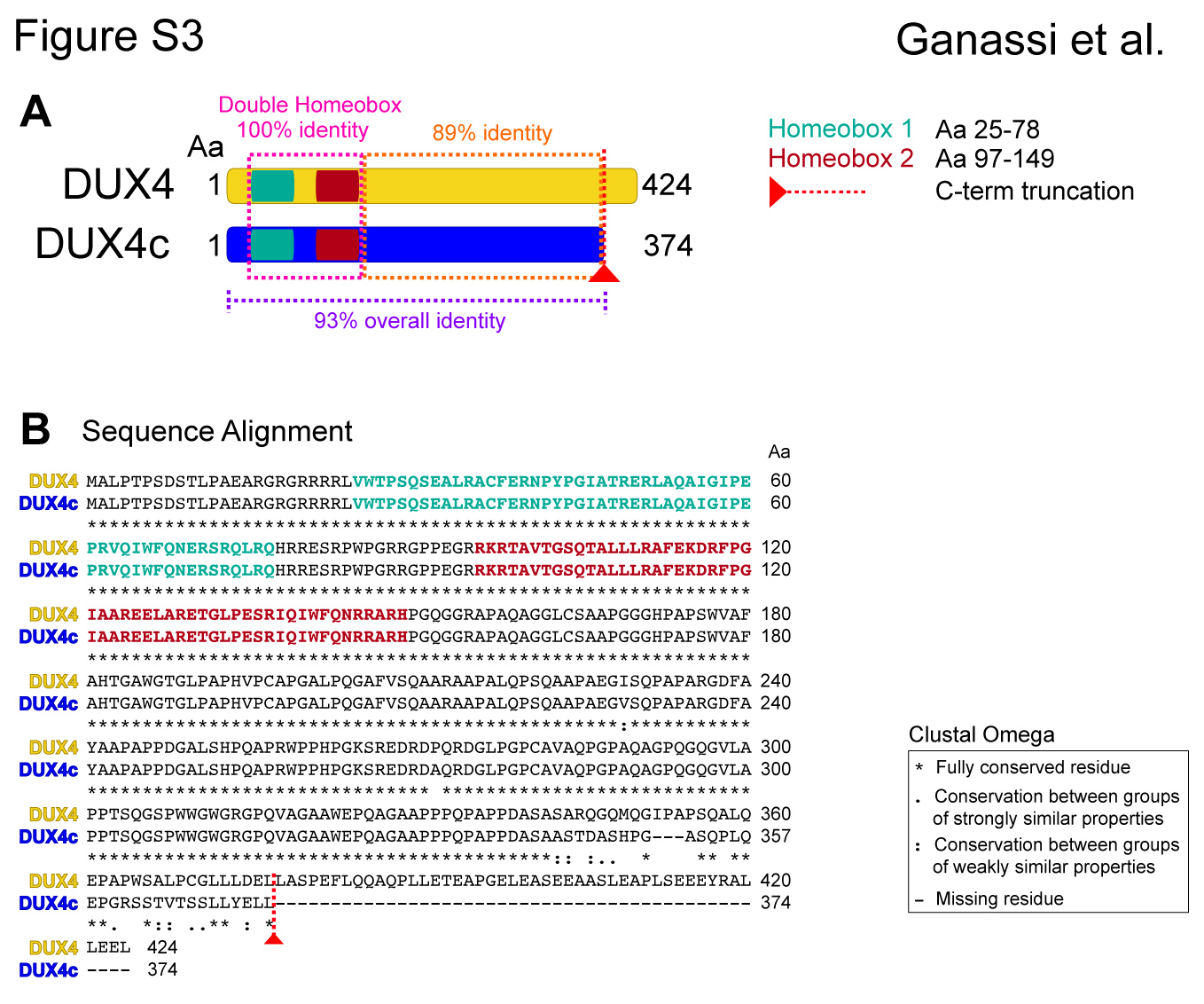

Supplement: Supplementary file 2 [file Image3.JPEG]

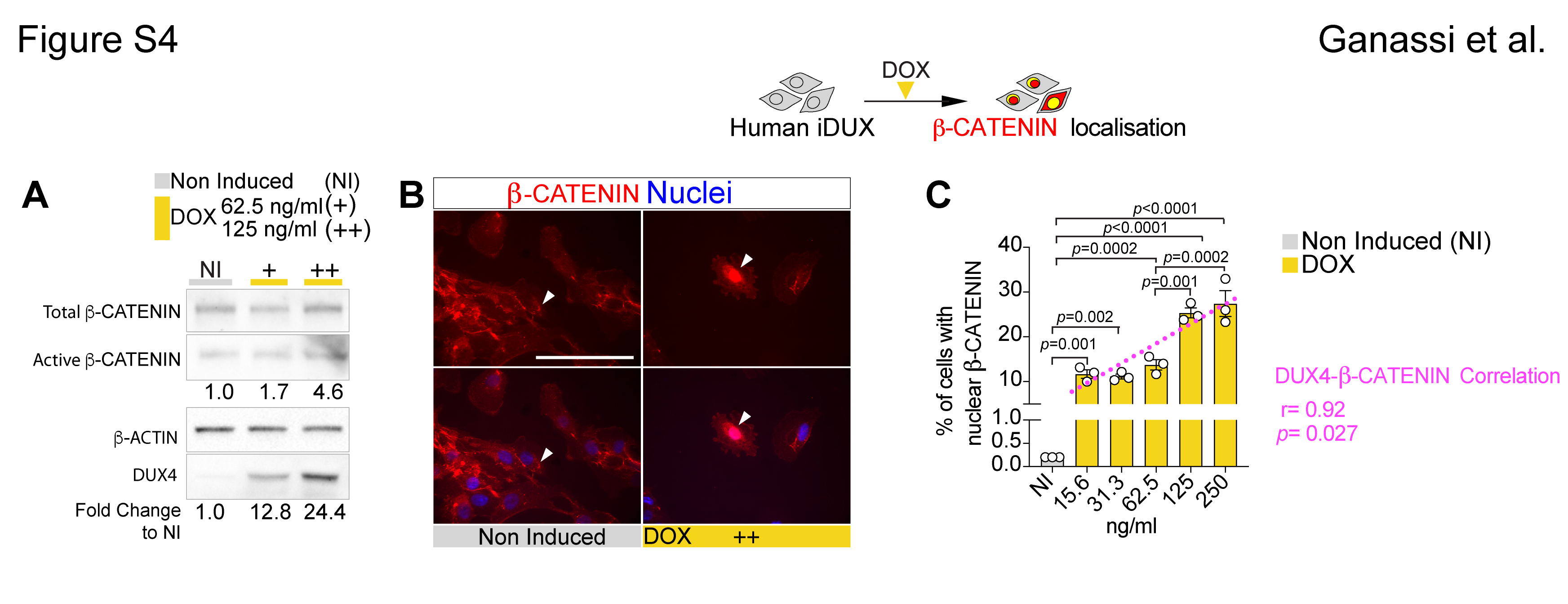

Supplement: Supplementary file 4 [file Image4.TIF]

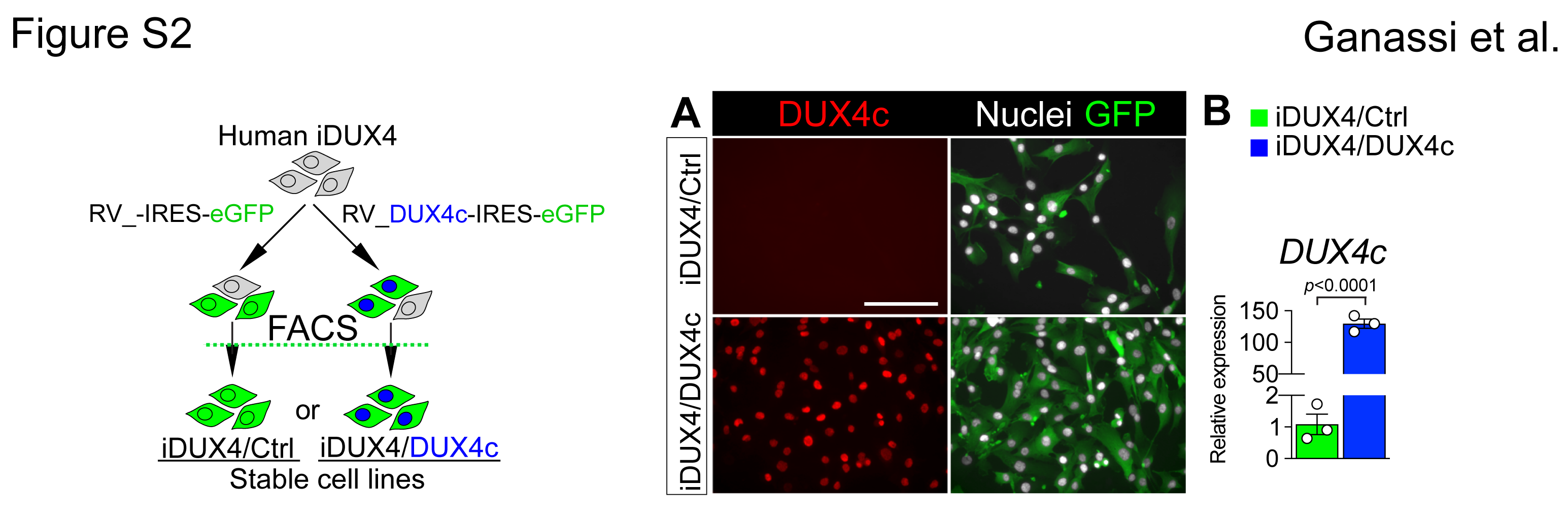

Supplement: Supplementary file 5 [file Image2.TIF]

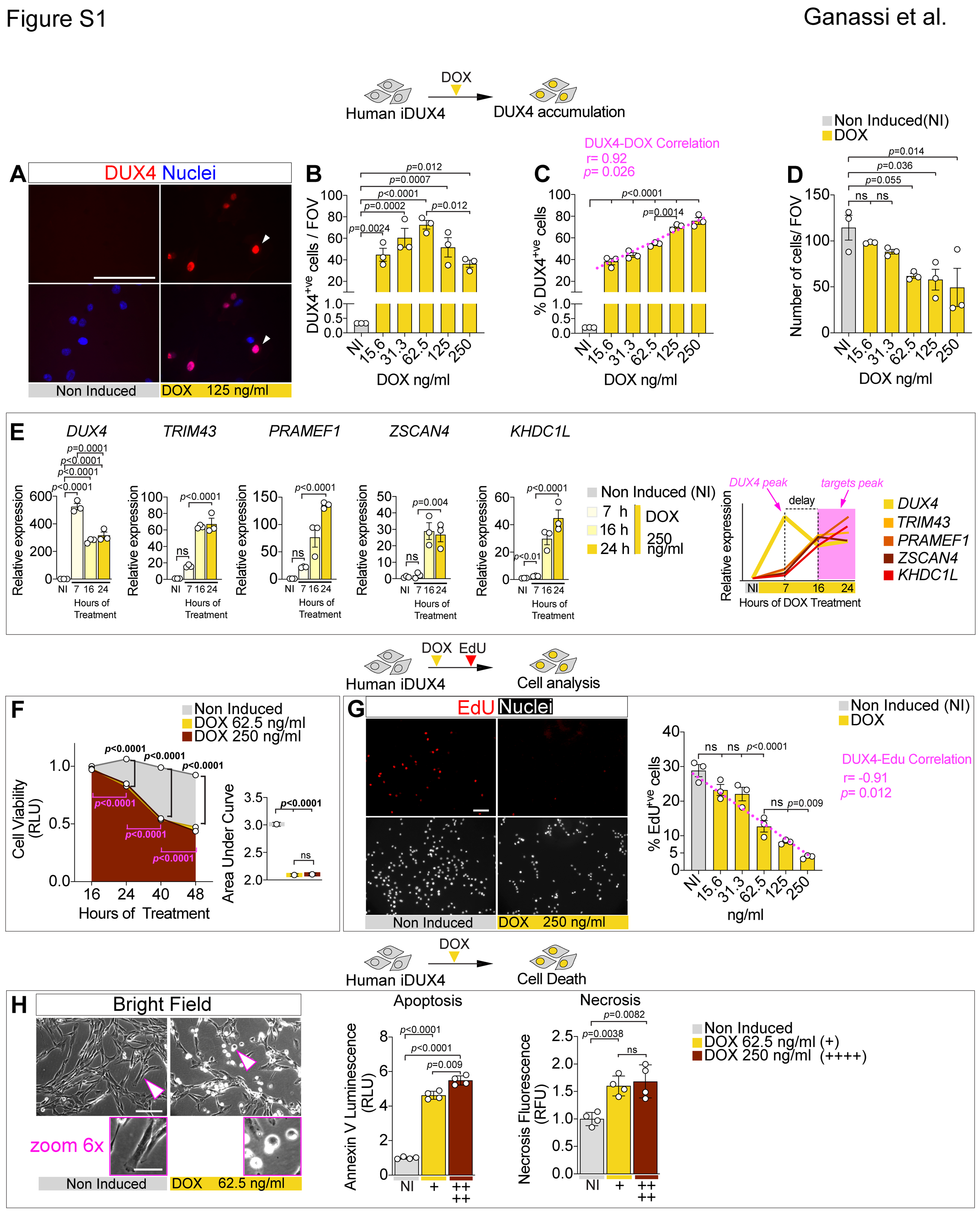

Supplement: Supplementary file 6 [file Image1.TIF]

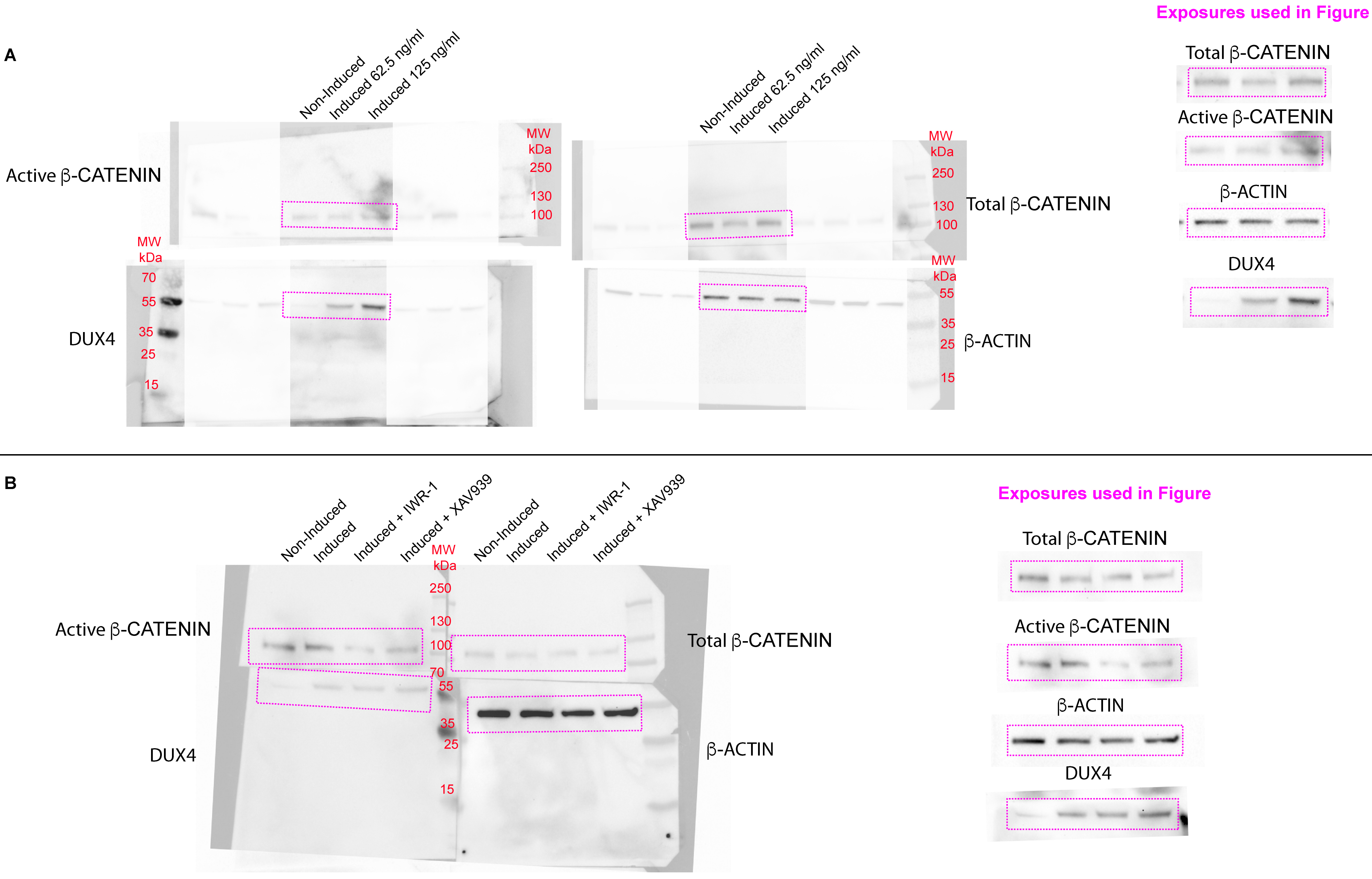

Supplement: Supplementary file 8 [file Image7.jpg]

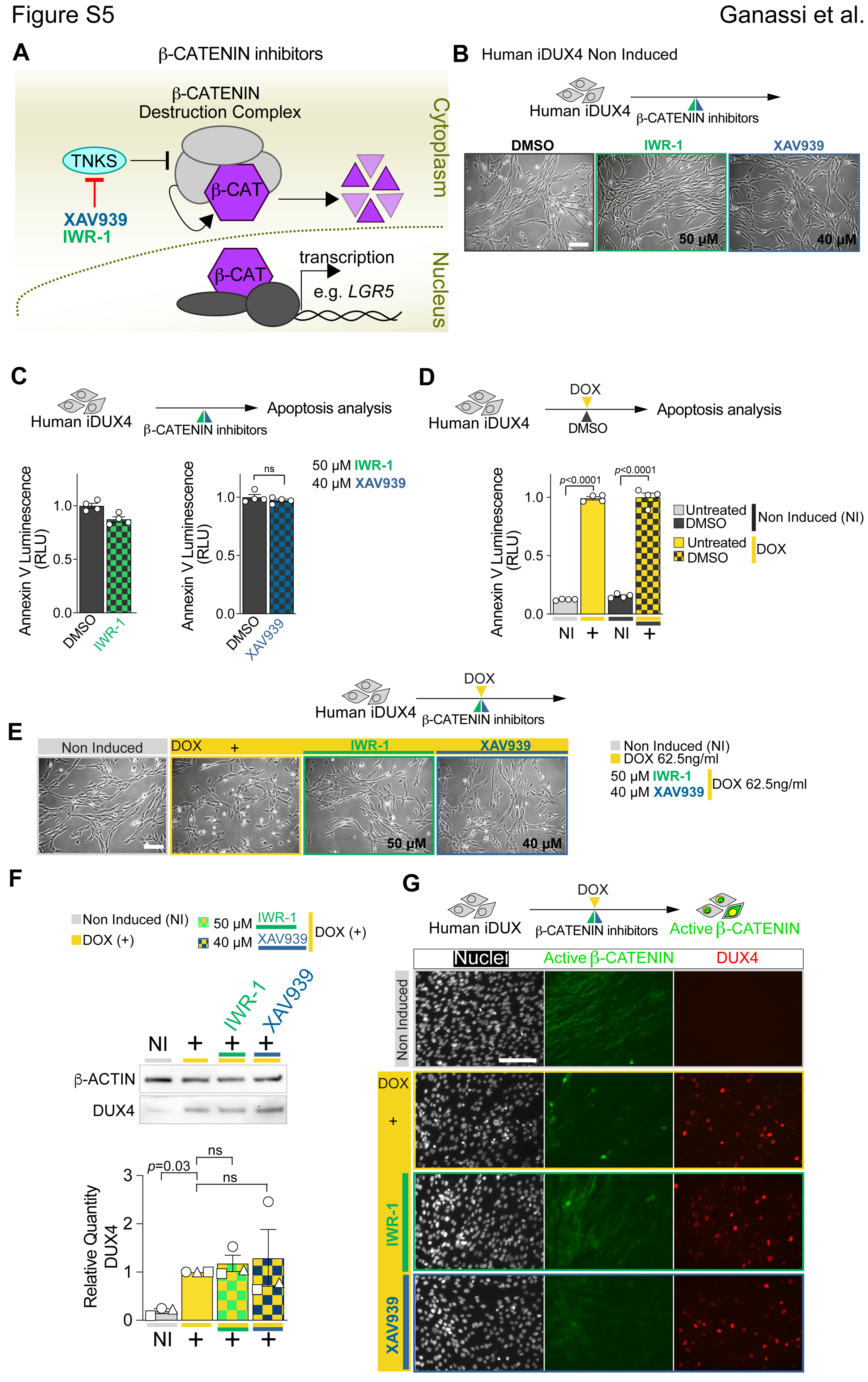

Supplement: Supplementary file 10 [file Image5.TIF]

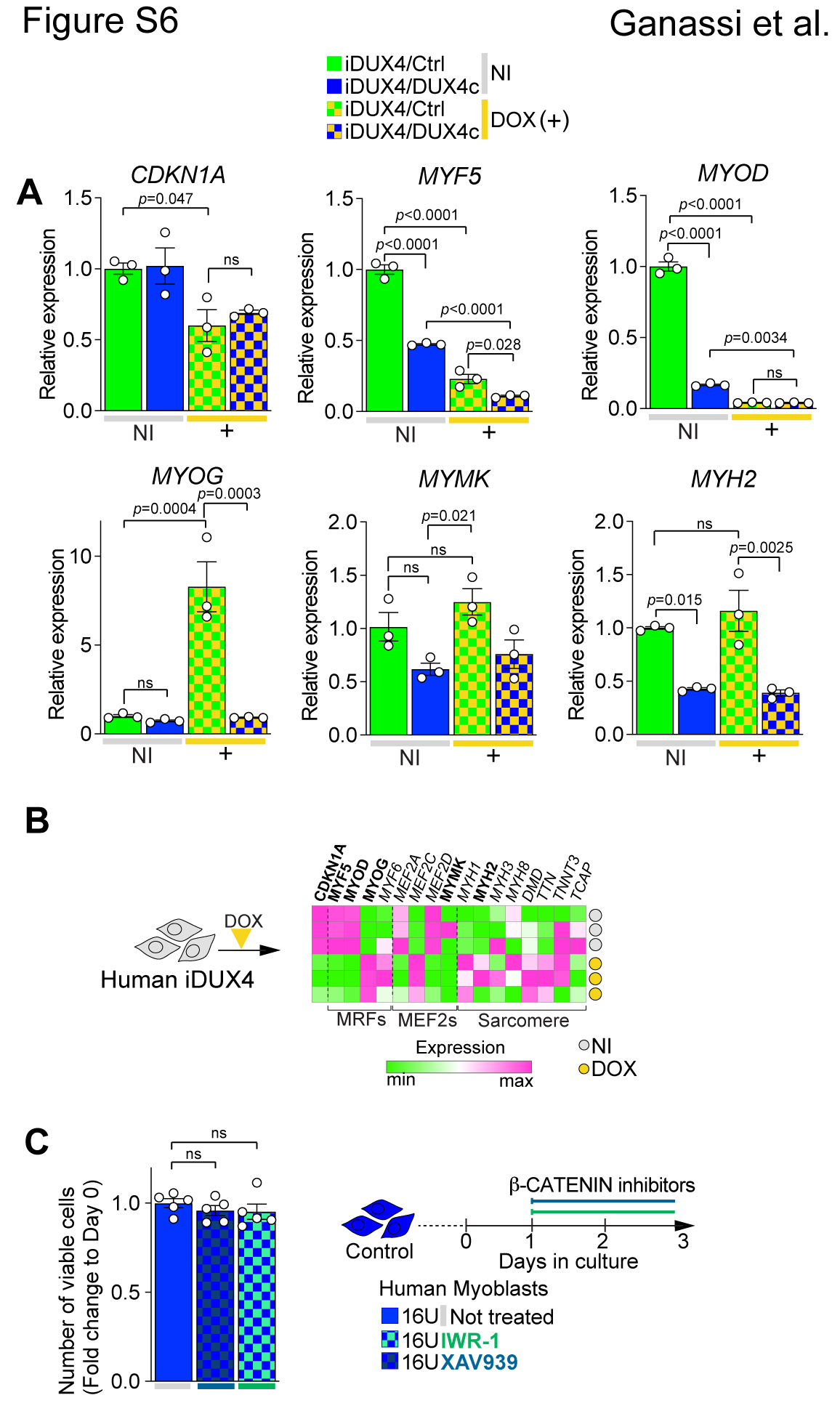

Supplement: Supplementary file 11 [file Image6.JPEG]
